# Molecular Mechanisms Underlying Neuroinflammation Elicited by Occupational Injuries and Toxicants

**DOI:** 10.3390/ijms24032272

**Published:** 2023-01-23

**Authors:** Dhruba Pathak, Krishnan Sriram

**Affiliations:** Health Effects Laboratory Division, National Institute for Occupational Safety and Health, Morgantown, WV 26505, USA

**Keywords:** Alzheimer’s disease, amyotrophic lateral sclerosis, astrocytes, cell signaling, gliosis, hydrocarbons, inflammation, immune response, metals, microglia, multiple sclerosis, nanoparticles, neuroinflammation, neurological disorders, neurodegenerative diseases, occupational injury, Parkinson’s disease, traumatic brain injury, workplace toxicants

## Abstract

Occupational injuries and toxicant exposures lead to the development of neuroinflammation by activating distinct mechanistic signaling cascades that ultimately culminate in the disruption of neuronal function leading to neurological and neurodegenerative disorders. The entry of toxicants into the brain causes the subsequent activation of glial cells, a response known as ‘reactive gliosis’. Reactive glial cells secrete a wide variety of signaling molecules in response to neuronal perturbations and thus play a crucial role in the progression and regulation of central nervous system (CNS) injury. In parallel, the roles of protein phosphorylation and cell signaling in eliciting neuroinflammation are evolving. However, there is limited understanding of the molecular underpinnings associated with toxicant- or occupational injury-mediated neuroinflammation, gliosis, and neurological outcomes. The activation of signaling molecules has biological significance, including the promotion or inhibition of disease mechanisms. Nevertheless, the regulatory mechanisms of synergism or antagonism among intracellular signaling pathways remain elusive. This review highlights the research focusing on the direct interaction between the immune system and the toxicant- or occupational injury-induced gliosis. Specifically, the role of occupational injuries, e.g., trips, slips, and falls resulting in traumatic brain injury, and occupational toxicants, e.g., volatile organic compounds, metals, and nanoparticles/nanomaterials in the development of neuroinflammation and neurological or neurodegenerative diseases are highlighted. Further, this review recapitulates the recent advancement related to the characterization of the molecular mechanisms comprising protein phosphorylation and cell signaling, culminating in neuroinflammation.

## 1. Introduction

Neuroinflammation is characterized by a complex inflammatory process in the CNS, a biological response of the neuroimmune system elicited by various chemical, biological, and physical agents. However, little is known about how neurons, glia, and resident macrophages interact during the inflammatory cascade, thereby inducing disease pathogenesis. Remarkably, inflammation is an evolutionarily conserved process during the activation of the immune and non-immune cells against an antigen or tissue injury [1,2,3]. It has been speculated that acute or intermittent inflammation is critical for survival during injury and infection, but chronic systemic inflammation can be counterproductive [3,4,5]. 

There is scientific consensus that certain environmental and social influences, as well as lifestyle factors, can contribute to chronic systemic inflammation, which leads to several disease outcomes such as autoimmune, cardiovascular, hepatic, renal, cancer, diabetes, as well as neurodegenerative disorders, all of which are significant global public health issues [3,4,5]. 

An inflammatory response occurs following infection or after an injury caused by physical, chemical, or metabolic insults. Inflammation elicited by systemic infection is mediated by pattern recognition receptors expressed on innate immune cells, which recognize conserved molecular motifs on microbes/pathogens, called pathogen-associated molecular patterns (PAMPs) [3]. Similarly, inflammation triggered by physical, chemical, or metabolic insults are mediated by damage-associated molecular patterns (DAMPs), also known as alarmins, which are endogenous distress molecules released by stressed, injured, damaged, and necrotic cells [3]. Like PAMPs, DAMPs can also activate innate immune responses. Evidence from preclinical studies suggests that acute and chronic inflammatory outcomes are associated with the release of proinflammatory cytokines and chemokines, which modulate the secretion or release of various hormones (e.g., gonadotropin-releasing, follicle-stimulating, and luteinizing) and neurotransmitters (e.g., norepinephrine, dopamine, and acetylcholine) [6]. However, the unresolved issue is the determination of how inflammatory mediators exert their effects on the brain, and if there are region-specific differences in the inflammatory profile, response, and injury outcomes.

An interesting aspect of a recent study is the recognition that the brain parenchyma lacks memory T-cells, which makes the inflammatory process quite different from the inflammation occurring in other organs of the body. Outside the brain, memory T-cells, the antigen-specific T cells that persist for long periods after infection, are central to orchestrating antigen-specific responses to repeated infectious exposures [7]. A subgroup of circulating memory T-cells has been shown to enhance inflammation by migrating to the sites of injury or by making their entry into lymph nodes to initiate cellular immune responses, including the production of additional effector T-cells, in lymphoid tissues [7]. Infectious and non-infectious agents, as well as cellular damage signals, can activate inflammatory cells through the nuclear factor kappa B (NF-κB), janus kinase-signal transducer and activator of transcription (JAK-STAT), and the mitogen-activated protein kinase (MAPK), signaling pathways [4]. 

However, within the brain, “microglia,” the resident macrophages, play a vital role in the occurrence and development of neurological dysfunction, including multiple sclerosis (MS), ischemic brain injuries, and Alzheimer’s disease (AD) [8,9,10]. Several studies have indicated that microglial polarization can activate the astrocytic function, which produces proinflammatory markers [11,12]. Microglial polarization into either M1 (pro-inflammatory) or M2 (anti-inflammatory) phenotype, which is termed classical activation and alternative activation, respectively, occurs due to perturbation in the microglial micro-environment [13]. Microglia are key regulators of neurogenesis and are known to control the number of neuronal precursor cells [14], as well as participate in the formation and elimination of neuronal synapses [15]. In the CNS, microglia serve as the main antigen-presenting cells and mediate autoimmune effector T-cell infiltration to the brain, as seen in MS. 

When microglia are activated, their cell body enlarges, their processes become shorter or withdrawn, and they assume a round or amoebic shape, which allows for their migration to sites of injury and gain phagocytic abilities [9,16]. Activated microglia express microglial receptors, including the triggering receptor expressed on myeloid cells-2 (TREM2), low-density lipoprotein receptor-related protein 1 (LRP1), toll-like receptors 2 and 4 (TLR2 and TLR4), cholinergic receptor nicotinic alpha 7 subunit (CHRNA7; also known as alpha 7 subtype of nACHR/α7nACHR), and the calcium-sensing receptor (CASR) [10]. It is now known that astrocytes are not well equipped with receptors recognizing pathogens compared to microglia [17,18]. When activated by polarized microglia, they become reactive, release inflammatory mediators, and modulate inflammation.

Industrial chemicals such as aldehydes, polycyclic aromatic hydrocarbons, phenols, phthalates, per- and polyfluoroalkyl- substances, pesticides, metals, and others, can cause inflammation via multiple mechanisms [3,19] including endocrine disruptors or cytotoxic agents. These chemicals have been associated with diverse diseases such as hormone-dependent cancers, metabolic syndrome, type 2 diabetes, hypertension, cardiovascular disorders, respiratory diseases, autoimmune diseases, and neurodegenerative disorders [3]. Additionally, metal toxicants such as aluminum (Al), copper (Cu), zinc (Zn), and iron (Fe) deposits have been reported within the core and periphery of senile plaques and colocalize with beta-amyloid, suggesting their role in neurodegenerative diseases such as AD [20].

This review aims to summarize the recent research highlighting the role of occupational toxicants in neurodegenerative diseases, with a specific focus on gliosis, including its genetic, molecular, signaling, and neuroinflammatory features.

## 2. Occupational Brain Injuries

An occupational injury describes any injury or illness to a worker as related to their specific work/occupational demands or requirement [21]. It is often a life-altering event that is also considered a form of disability [22], which is covered under the provisions of the 1990 Americans with Disabilities Act. The National Safety Council identified slips, trips, and falls are among the top three leading causes of work-related injuries [23]. Slips or trips causing a fall are prevalent work-related injuries accounting for 20% to 40% of disabling occupational injuries [21,24]. 

The National Institute for Occupational Safety and Health (NIOSH) has studied slips, trips, and falls in healthcare settings, and wholesale/retail trade establishments to identify risk factors [25,26]. The common risk factors that can cause slips, trips, and falls at the workplace include liquid spills, ice, snow, rain, loose flooring, carpets, mats or rugs, boxes/containers, poor or improper lighting, work pressure, age, fatigue, failing eyesight, and inappropriate/poorly fitting footwear [25,26,27]. Traumatic workplace injuries can result in protracted disability and other health outcomes, which are the primary causes for a prolonged delay in return to work of the injured worker [28]. 

In a variety of workplaces, slips, trips, and falls can be one of the primary causes associated with traumatic brain injury (TBI). Notably, falls account for almost half of the emergency department visits for TBIs. Severe blunt forces, e.g., bumps and blows, which cause hits against the skull, can also perturb brain function, causing damage [5]. The injury can be penetrating as caused by a sharp object or non-penetrating when hit by objects/projectiles. TBI can also occur through high-velocity probes or projectiles such as gunshots and shrapnel. An explosion can cause concussions and TBI. Different types and grades of TBI common in occupational injuries include mild, moderate, severe, uncomplicated, complicated, closed, open, and non-traumatic. Specific occupations likely to have a higher risk of TBIs include construction and manufacturing, healthcare, hospitality and service industry, military, and law enforcement [29,30].

In addition to slips and falls, chemical exposures are also occupational hazards. For example, volatile organic compounds (VOC) that are generally released from paints, varnishes, waxes, and solvents used in the paint, automobile, furniture, and electronic manufacturing industries have been shown to cause neurological problems. They are organic compounds with a variety of functional groups and include xylenes, toluene, formaldehyde, and benzene, all of which have been reported to be environmental hazards and cause serious occupational injuries [19]. VOC have been shown to interact with nitrogen oxides (NOx), forming ozone and peroxyacetyl nitrate, which are known occupational and environmental toxicants [19]. Little is known about the mechanism by which VOC induces inflammation. Nevertheless, VOC are known to impact the cellular machinery, including transcriptional regulation of CD4 T-cell related genes, demethylation, the release of cytokines, activation of various T-helper cells (Th1, Th2, Th17, and Treg), the release of adhesion molecules (CTLA4, CD40L, and CD70), upregulation of cell cycle proteins (CDKN1A), and triggering autoimmune diseases [31]. Notably, VOC found in the air as pollutants have been linked to neurological diseases, including stroke, PD, and AD because of their ability to easily cross the blood-brain barrier (BBB) and activate the CNS response [19].

Another prominent industrial chemical that is known to enter the CNS and exert its toxic actions is 1,2-Dichloroethane (DCE). DCE is a synthetic chemical widely exploited in the production of vinyl chloride, which in turn is the starting material for a variety of plastic and vinyl products. DCE-based vinyl products are extensively used in furniture and automobile upholstery, wall coverings, housewares, and automobile parts. Clinical investigations and post-mortem examinations of the brains of workers exposed to DCE revealed that brain edema was the primary pathological change leading to death among DCE-exposed workers [32,33]. Similarly, in DCE-intoxicated mice, astroglial and microglial cross-talk caused inflammation and brain edema [34]. DCE is known to easily cross the BBB and target glial cells, particularly the astroglia and oligodendroglia [35]. Recently, DCE has been shown to inhibit the expression of aquaporin 4, a brain-specific water channel protein, in astrocytes, and reduce myelin basic protein in oligodendrocytes leading to demyelination [36]. 

## 3. Reactive Gliosis in Neural Injury and Neurodegeneration 

Gliosis, a “reactive” state of glia, is a pathological hallmark of all types of central nervous system (CNS) injuries [37,38]. The activation of astrocytes and microglia is the universal component of the neuroinflammatory response. It is implicated in toxicant-induced neurotoxicity and progression of neurodegeneration after ischemia, seizure, AD, PD, MS, and ALS, to name a few [37,39,40]; see Table 1 and Table 2. 

Responses to injury and disease in the CNS involve the interaction of various neural and non-neural cells to maintain cellular homeostasis, neuronal integrity, neuronal viability, and neuronal function [126]. 

Some fundamental issues must be considered to fully understand the involvement of glial response in toxicant-induced neurotoxicity when measuring the expression of glial markers. First, the number of glial cells (astroglia and microglia) can vary across brain regions; second, glial cells from different brain regions exhibit a differential time course of expression; and third, the magnitude of glial response to an insult can vary [37,127]. Another consideration is that the glial response to toxicant exposure can vary depending on the injury stage or disease progression in which they occur.

Astrocytes and microglia adopt an activated phenotype following an insult, resulting in two polarization states, the pro-inflammatory phenotypes (A1 and M1, respectively) and an anti-inflammatory phenotype (A2 and M2) [128]. Accumulated scientific evidence also suggests that astrocytes and microglia express endogenous pattern recognition receptors including a variety of DAMPs, which can result in a molecular signaling cascade toward inflammation and disease propagation [39,129]. Activation of DAMP-mediated cellular signaling is known to induce the secretion of numerous pro-inflammatory mediators including adipokines, cytokines (for example, IL1B, IL6, TNFA), and chemokines (C-C motif chemokine ligand 2, CCL2/MCP1), as well as activating various cell types such as adipocytes, endothelial cells, and resident immune cells [3]. In the pathological brain, stretch injury to the astrocyte causes the release of vasoactive factors such as endothelins and isoprostanes, upregulation of the inositol triphosphate signaling, and enhancement of neural cell sensitivity to extracellular glutamate and inflammatory cytokines [130]. 

Microglia are the primary resident sentinel immune cells that are thought to maintain homeostasis and one of the significant non-neuronal cell types that contribute to neurodegeneration [4,39,131,132], see Box 1. An acute insult can instigate microglial activation, causing elongation of its processes and increasing the expression of marker proteins such as AIF1/IBA1 and integrin subunit alpha M (ITGAM/CD11B) [133]. Olfactomedin-like protein 3 (OLFML3) has also been determined as microglia-specific gene that is involved in early developmental patterning, while sialic acid binding Ig-like lectin H (Siglec-H) is associated with innate immune cell differentiation [132,134,135]. During the presentation of cues such as foreign agents/particles, cell debris, or toxicants, the homeostatic resident microglia transform and attain a reactive state (see Box 1).

Box 1 **Homeostatic and Inflammatory Microglial Markers.**

**Homeostatic microglial markers**

- Purinergic Receptor P2Y12 (P2RY12)
- Transmembrane protein 119 (TMEM119)
- Olfactomedin-Like Protein 3 (OLFML3)
- Fc Receptor-Like 2 (FCRLS)
- Spalt Like Transcription Factor 1 (SALL1)
- C-X3-C Motif Chemokine Receptor (CX3CR1)
- G Protein-Coupled Receptor 34 (GPR34)

**Inflammatory microglial markers**

- Galactose-Specific Lectin 3/Galectin 3 (LGALS3)
- C-Type Lectin Domain Family 7 member A (CLEC7A)
- Allograft Inflammatory Factor 1/Ionized Calcium-Binding Adapter Molecule 1 (AIF1/IBA1)
- Transmembrane Immune Signaling Adaptor TYROBP/TYRO Protein Tyrosine Kinase Binding Protein (TYROBP)
- Tumor necrosis factor alpha (TNFA)
- C-C Motif Chemokine Ligand 2 (CCL2/MCP1)
- Integrin Subunit Alpha M (ITGAM)

### 3.1. Gliosis in Brain Injury

TBI is characterized as one of the leading causes of death and disabling occupational injuries [136,137,138], with a high incidence rate in both the military and civilian populations [139]. Nearly 5.3 million people live with TBI in the U.S. [136,137,140,141,142], including work-related TBI. Approximately one in four mild TBI (mTBI) cases in adults are considered work-related [28]. However, neuroinflammatory and neurological outcomes of work-related TBI have not been thoroughly investigated. The U.S. Centers for Disease Control and Prevention (CDC) has disseminated the case definitions for TBI using the Ninth and Tenth Revisions of the International Classification of Disease, Clinical Modification (ICD-9-CM and ICD-10-CM codes), commonly used in injury research [140,143]. Similarly, the Occupational Injury and Illness Classification System were developed by the U.S. Bureau of Labor Statistics to characterize work-related injuries and illnesses.

All-encompassing, the terms concussion, mild head injury, mTBI, and cerebral trauma, are used interchangeably to describe the physical damage and the ensuing symptomatic consequences arising from impairment of the brain structure or function [5,144]. Concussions are usually caused by a blow, bump or jolt to the head. A hit to the body that causes the head and brain to rush back and forth can also cause a concussion [145].

Immune cell activation and inflammatory responses are the cardinal features of traumatic injury. However, poor or failed resolution of acute inflammation, and compromise of the defense mechanisms, can contribute to chronic inflammation or an exaggerated systemic inflammatory response syndrome (SIRS) [138]. SIRS occurs shortly after trauma or traumatic injury when inflammatory cytokines enlist peripheral leukocytes to the site of the inflamed or injured tissue [138,146].

Several lines of evidence point to a role for inflammation in the clinical and functional outcomes seen in TBI [5]. Inflammatory mediators, e.g., cytokines and chemokines released during traumatic injury can augment the immune response by engaging several immune cells. Both human and animal studies have shown an increase in the expression of high mobility group box 1 protein (HMGB1) following brain injury [5], which binds to TLR4 and initiates the inflammatory cascade. The deposition of oxidized phospholipids and oxygenated free-fatty acids has also been shown to cause acute brain injury and induce an inflammatory response [147]. In addition to changes in the lipid profile, several other genes are also upregulated following mild and severe TBI. These include antigen-presenting factors (MHC-II, CD86, CD74), cytokines (IL6, IL12, IL10, IL1B, TGFB, IFNG), phagocytosis factors (FCGR, FCGR4, C3, C4), chemotaxis factors (CCL2, CCL4, CXCL1, CXCL4), and astrocytic proteins (GFAP, AQP4) [5].

Head injury is a significant risk factor for the initiation and progression of dementia, characterized by the aggregation of beta-amyloid (Aβ) plaques [148]. In addition, repeated insults/injuries to the brain heighten the glial response by enhancing microglial or astrocytic cell density and release of inflammatory mediators [149,150]. Evidence obtained from human post-mortem tissues revealed that repeat injuries generate a more robust glial response, demonstrating that phosphorylated tau and CD68 cell density can be predictors of repeated head injury [151].

The primary pathological outcomes associated with TBI have been reported to include focal intracranial hemorrhage, epidural and subdural hematoma, and axonal injury [139]. Computed tomography studies also provided evidence of meningeal vascular leakage in approximately 50% of concussed patients who were otherwise considered clinically normal [152]. Such vascular changes can potentially lead to secondary lesions that can progressively culminate in neurological or neurodegenerative outcomes. 

**Figure 1:**
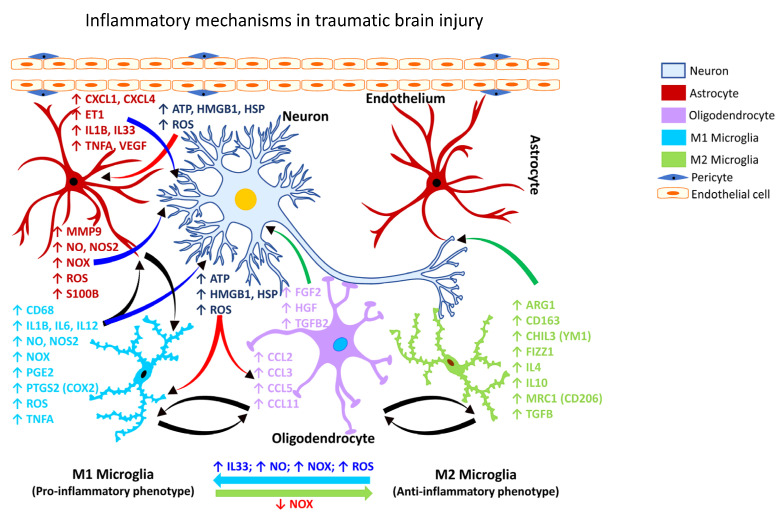
Inflammatory mechanism in traumatic brain injury. Schematic representation of the molecular mechanisms associated with glia-mediated functional interactions and systematic perturbations within the CNS to induce neuroinflammation in traumatic brain injury. The endothelial cells form the inner lining of the blood vessel with pericytes enveloping the surface of the vasculature forming tight junctions to maintain the BBB integrity. Upon insult, neurons release danger/damage signals that cause activation of neighboring glial cells. M1 microglia (proinflammatory phenotype, neurotoxic) release various proinflammatory mediators including free radicals, cytokines, and chemokines that further stimulate other glial cells and collectively contribute to exacerbating the neuronal injury/damage. M2 microglial cells (anti-inflammatory phenotype, neuroprotective) can polarize to an M1 state and release proinflammatory mediators in the presence of increased levels of NOX, ROS, NO, and IL33 released by M1 microglia and/or astrocytes and thereby augment the neuroinflammatory and neuronal injury process leading to synaptic dysfunction, neuronal injury, and neuronal death. Astrocytes respond by releasing proinflammatory mediators including free radicals, cytokines, and chemokines, which further contribute to enhancing the endothelial permeability, disrupting BBB integrity, and allowing for infiltration of peripheral immune cells, events that further intensify inflammation and neuronal injury. Feedback regulation of NOX or its inhibition causes M1 microglia to polarize to the M2 state (anti-inflammatory phenotype), which downregulates M1 functions and promotes regulation of neuroinflammation and neurorepair by releasing anti-inflammatory mediators, e.g., cytokines, neurotrophic, and growth factors. Mediators released by specific neural cell types (neuron, astrocyte, microglia, or oligodendrocyte) are listed adjacent to each cell type in similar colored text. Curved arrows indicate the direction of signal flow between various neural cells for the inflammation activation process. Red curved arrows show the directional flow of danger/damage signals from neurons to glial cells (astroglia, microglia, oligodendroglia); blue curved arrows show the flow of proinflammatory signals from astrocytes and M1 microglia towards distressed neurons; green curved arrows show the flow of neurotrophic signals from M2 microglia and oligodendroglia towards the distressed neurons as a neuroprotective/neurorescue endeavor; black curved arrows show the directional crosstalk among various glial cells to mount a glial response to neuronal injury/damage. ↑, increase; ↓, decrease; ARG1, arginase 1; ATP, adenosine triphosphate; BBB, blood-brain barrier; CCL2, C-C motif chemokine ligand 2 (also referred to as MCP1, monocyte chemoattractant protein 1); CCL3, C-C motif chemokine ligand 3; CCL5, C-C motif chemokine ligand 5; CCL11, C-C motif chemokine ligand 11; CD68, CD68 molecule; CD163, CD163 molecule; CHIL3, chitinase-like protein 3 (also referred to as YM1); CXCL1, C-X-C motif chemokine ligand 1; CXCL4, C-X-C motif chemokine ligand 4; ET1, endothelin 1; FGF2, fibroblast growth factor 2; FIZZ1, found in inflammatory zone 1; HGF, hepatocyte growth factor; HMGB1, high-mobility group box 1; HSP, heat shock proteins; IL1B, interleukin 1 beta; IL4, interleukin 4; IL6, interleukin 6; IL10, interleukin 10; IL12, interleukin 12; IL33, interleukin 33; MMP9, matrix metalloproteinase 9; MRC1, mannose receptor C-type 1 (also referred to as CD206); NO, nitric oxide; NOS2, nitric oxide synthase 2 (inducible nitric oxide synthase); NOX, NADPH oxidase 1; PGE2, prostaglandin E2; PTGS2, prostaglandin-endoperoxide synthase 2 (also referred to as COX2, cyclooxygenase 2); ROS, reactive oxygen species; S100B, calcium binding protein B; TGFB, transforming growth factor beta; TGFB2, transforming growth factor beta 2; TNFA, tumor necrosis factor alpha; VEGF, vascular endothelial growth factor.

Following TBI, activated microglia/macrophages exhibit different phenotypic features [153], commonly referred to as classically activated or M1 (pro-inflammatory) and alternatively activated or M2 (anti-inflammatory) microglia (Figure 1). The inflammatory or anti-inflammatory glial response to TBI is governed by numerous cell-signaling events that are selectively activated depending on the form and severity of the neural insult [126,154,155]. Thus, microglia become rapidly activated in response to CNS injury caused by TBI.

In animal models of concussion brain injury, microglial activation, robust upregulation of inflammatory cytokines, and impairment of white matter have been shown to be the key pathological features [153]. Mounting evidence points to a progressive neuroinflammatory process after a head injury that is persistent even after the resolution of the acute injury response [156], which can potentially contribute to cognitive and behavioral deficits.

The caveats in TBI studies arise due to the complex immunological mechanisms and the interindividual differences in the pathological response among humans, which creates inconsistencies in translating findings from animal research into clinical applications. There are analytical constraints in identifying the precise mechanisms for primary and secondary injuries linked to TBI in experimental models, which need to be singled out to target-specific subgroups for better translation toward injury diagnosis and disease intervention in humans [5]. The secondary inflammation associated with TBI can be slower in development ranging from months to years, which could further impede the translational applications. TBI can be driven by several cellular signaling pathways involving free radicals, membrane damage, immune activation, and excitotoxicity due to excess glutamate, among others [5,137,141,147].

### 3.2. Gliosis Following Chemical-Induced Neural Injury

#### 3.2.1. Gliosis Associated with Hydrocarbon Exposure

The rapid industrialization and urbanization have brought with it an unprecedented rise in human exposure to an ever-expanding list of hazardous chemicals and pollutants [157]. Every year, more than 2000 new chemicals are introduced into the environment, and humans can potentially come in to contact with such chemicals either at the workplace or through daily consumption or use, including via food, cosmetics, pharmaceuticals, cleaning agents, and herbicides/pesticides. In 2008, the Toxicology in the 21st Century (Tox21) Consortium [158], a collaboration of federal agencies, including the U.S. Environmental Protection Agency (EPA), the National Institute of Environmental Health Sciences (NIEHS)-National Toxicology Program (NTP), the National Center for Advancing Translational Sciences (NCATS), and the Food and Drug Administration (FDA) came into effect to evaluate and understand the adverse human health risks of commercial chemicals, including pesticides, food additives/contaminants, and medical products [159,160]. Some of the chemicals of interest to the Tox21 program are polycyclic aromatic hydrocarbons, phthalates, per- and poly-fluoroalkyl substances, bisphenols, and flame retardants [160], all of which have potential for adverse human exposures in occupational settings given the consumption demand and high production volumes.

Exposure to aliphatic hydrocarbons, particularly n-hexane and halogenated compounds, has been shown to cause a widespread dopaminergic neuronal loss in the substantia nigra and depletion of tyrosine hydroxylase immunoreactivity in the striatum, which was associated with severe gliosis [161,162]. The effects of several natural and synthetic compounds, including halogenated aromatic hydrocarbons (e.g., biphenyls, dioxins, furans) and polycyclic aromatic hydrocarbons (PAH; e.g., benzo[*a*]pyrene, benzanthracenes, 3-methylcholanthrene) are mediated by the aryl hydrocarbon receptor (AHR/AhR). The AHR is a ligand-dependent transcription factor that integrates various metabolic cues from the environment, food chain, or microorganisms to regulate transcription in a cell- and ligand-specific manner [163]. Increased AHR immunoreactivity is associated with activated microglia in the middle cerebral artery occlusion (MCAO) model of brain ischemia [164]. Evaluation of AHR immunoreactivity in human hippocampal post-mortem tissue and its association with reactive astrocytes revealed their greater participation in the development of neurodegenerative diseases, including AD [165], suggesting that occupational and environmental exposure to PAH may contribute to the neuropathogenesis seen in many neurodegenerative disorders.

Deliberate inhalation of certain volatile hydrocarbons for their mood-altering effects is prevalent among humans. Volatile hydrocarbons are predominantly found in glues, solvents, lighter fluids, gasoline, and paints. High exposure to PAH has been shown to reduce subcortical volume and cause cortical thinning in older people, primarily affecting the parietal, temporal, and insular regions in men, while in women, the frontal and parietal cortical lobes appear to be affected more severely [166]. Additionally, PAH formed during the incomplete combustion of organic matter poses a significant risk for firefighters at fire sites [167], indicating a potential occupational health risk.

Toluene is an aromatic hydrocarbon widely used in various occupational settings, including the paint and adhesive industry; rubber and lumber sector; dry cleaning; automobile and aviation manufacturing; and chemical industries. The highly lipophilic nature of toluene [168] can facilitate its entry into the brain after inhalation exposure and target the myelin sheath given it is made up of 70–75% lipids. Thus, the effect of toluene on glial cells, particularly on Schwann cells, oligodendrocytes, and astrocytes are more significant than on the neuronal population, as axonal integrity is relatively well preserved in toluene leukoencephalopathy [168]. Toluene also disrupts the differentiation of astrocyte precursors and decreases ATPase activity in astrocytes [169,170]. Indeed, altered astrocytic function and reactive astrogliosis have been documented in human cases of toluene abuse [171]. Toluene is also known to dose-dependently inhibit *N*-methyl-D-aspartate (NMDA)-mediated excitatory postsynaptic currents (EPSCs) and induce a delayed but persistent reduction in evoked or spontaneous alpha-amino-3-hydroxy-5-methyl-4-isoxazole propionic acid (AMPA)-mediated EPSCs [172]. In addition, toluene also inhibits the nicotinic acetylcholine receptor, which has a critical role in brain development [173]. In rats inhaling toluene, loss of hippocampal neurons and cerebellar Purkinje cells is reported [174], with the latter also causing ‘thinning’ of the cerebellar white matter [174]. Chronic or repeated toluene exposure has been shown to induce pro-oxidants, reduce antioxidants, and cause memory impairment [175].

#### 3.2.2. Gliosis Associated with Metal Exposure

With rapid industrialization, there is an increasing demand for metals. The high-volume production and use of metals are of significant concern for occupational safety due to potential worker exposure and consequent adverse health effects. Chronic workplace exposure to high-production volume metals such as iron (Fe), aluminum (Al), and manganese (Mn) are believed to be associated with an increased risk of neurodegeneration. Fe translocation to the brain is mediated by the transferrin receptor and the solute carrier family 11 member 2 (SLC11A2; also known as divalent metal transporter 1/DMT1 [176]. Its prooxidant characteristics elicit ROS generation via the Fenton reaction and Haber-Weiss reaction [177], which can subsequently mediate oxidative stress. Free Fe has been known to induce fibrillation and aggregation of alpha-synuclein (αSYN; encoded by the gene SNCA/PARK1) in a dose- and time-dependent manner in cultured cells [178]. Emerging evidence also shows that Fe accumulation in the brain accelerates disease progression in AD, but the mechanism through which this occurs is not known [179]. Further, the study demonstrated the occurrence of Fe-accumulating microglia that exhibited a dystrophic morphological state. This subset of Fe-accumulating microglia showed augmented expression of the iron storage protein ferritin light chain (FTL) and allograft inflammatory factor 1 (AIF1; also known as ionized calcium-binding adaptor protein 1/IBA1) while downregulating the expression of transmembrane protein 119 (TMEM119) and the purinergic receptor P2Y12 (P2RY12) [179].

Al is a potent neurotoxic element involved in the etiology of occupational neurodegenerative disorders. Al causes oxidative stress leading to the deposition of intracellular reactive oxygen species [180]. However, the evidence suggesting the role of Al in the development of AD is inconsistent. Chronic oral administration of Al has been shown to increase Aβ levels in the cortex and hippocampus of rats [181]. Evidence shows that acute and chronic Al intoxication induces astrogliosis in the motor cortex and the hippocampus [180]. Exposure of Tg2576 mice, a model for AD, to Al resulted in increased expression of miRNAs (miR146a and miR125b) demonstrating a proinflammatory response like that seen in the brain of patients with AD [182,183,184].

Mn has been implicated in regulating cellular homeostasis and maintaining physiological functions. While Mn is an essential trace element for normal brain development and function, excess brain Mn is known to be neurotoxic. Occupational exposure to elevated airborne levels of Mn in mining and ferroalloy industries has been reported to cause neurological disorders [185,186,187,188,189,190,191], and has been linked to the slowed movement of upper extremities, poor balance and gait coordination, neuropsychological abnormalities, disruption of sleep, cognitive deficits, as well as parkinsonism [192,193,194]. Sustained exposure to low concentrations of Mn has been found to cause Mn-induced parkinsonism [195]. Microglial activation has been observed in the substantia nigra pars reticulata (SNpr) and substantia nigra pars compacta (SNpc) of cynomolgus macaques chronically exposed to Mn [196]. 

While the exact mechanisms of Mn transport and neurotoxicity are unclear, several studies suggest that Mn influx to the brain is carrier-mediated [197,198,199,200]. Other studies suggest that divalent metal transporters and L-type calcium channels may potentially be involved in the influx of Mn ions across the BBB [201,202]. Additionally, brain accumulation and toxicity of Mn is thought to be influenced by its elemental speciation. The oxidation state of Mn appears to be critical for its solubility, function, transport, retention, and toxicity [203,204]. Mn aerosol in ferroalloy industries is reported to exist in mixed oxidation states [205] including Mn (0), Mn (II), Mn (III) and Mn (IV). Of these, Mn (II) and Mn (III) are thought to be the predominant forms transported to the brain, where they potentially accumulate [206]. 

Welding fumes (WF) are a complex mixture of toxic metals and gases arising due to the burning of welding electrodes during welding. WF aerosols contain Mn, which is implicated in the development of PD-like neurological dysfunction seen among welders. Particles and aerosols inhaled through the nose and air passages can translocate to the brain via retrograde transport across olfactory neurons and accumulate in deeper brain areas [207,208,209,210]. WF may likely follow the same portals for entry into the brain. Alternatively, following deposition in the pulmonary targets, WF particles or soluble metal components of the WF may enter the systemic circulation and be transported to the brain following permeation through the BBB or the circumventricular organs (pituitary gland, median eminence, area postrema, choroid plexus), areas of the brain that are devoid of BBB. Indeed, there is evidence linking Mn and WF exposure to manganism and PD-like manifestations, including neuropsychological and neuropsychiatric disturbances [192,193,211,212,213,214,215,216,217,218,219]. Further, experimental studies have also shown that WF causes dopaminergic neurotoxicity [57,104,220,221].

#### 3.2.3. Gliosis Associated with Nanoparticles/Nanomaterials

In recent years, accumulating experimental evidence suggests the potential role of nanoparticles (NPs) in neuroinflammation and brain injury [222,223,224,225]. The toxicological effects of nanoparticles depend on their physicochemical properties such as size, shape, and surface charge. The underlying mechanisms of their toxicity are not fully realized, given this is an emerging area of research. Nonetheless, it is thought that much of their effects may be related to their physical interaction with cellular membranes, likely causing membrane disruption, eliciting inflammation, and generating free radicals, including reactive oxygen species (ROS) [226].

##### Carbon-Based Nanomaterials

Carbon nanotubes (CNTs) have the structure of tubes made of carbon-based nanomaterials. Carbon nanotubes find application in manufacturing nanocomposites and semiconductors because of their remarkable physicochemical properties, such as high flexibility, good thermal conductivity, low density, and high chemical stability [227]. Carbon nanomaterials exhibit a wide variety of toxic effects including inflammatory effects on dendritic cells, ROS generation, DNA damage, pulmonary macrophage activation and inflammation, lysosomal damage, mitochondrial dysfunction, and apoptosis or necrosis-mediated cell death [228]. Engineered carbon nanomaterials such as single-walled carbon nanotubes, double-walled carbon nanotubes, and multi-walled carbon nanotubes (MWCNT) have the potential to elicit neurotoxicity due to their small size, ability to aerosolize, and bio-persistence [229]. Inhaled MWCNT is distributed to various organs, including the brain [230]. Research has shown that MWCNT exhibits spatial association with Aβ fibrils in the brains of mice [231]. Further, long-term inhalation of MWCNT has been shown to cause neurological effects, including ROS production, lipid peroxidation, cytochrome *c* release, and mitochondrial swelling [224]. 

##### Metal Oxide Nanoparticles

Metal oxide nanoparticles (NPs), e.g., copper oxide NP (nano-CuO), iron oxide NP (nano-FeO), silica dioxide NP (nano-SiO_2_), titanium dioxide NP (nano-TiO_2_), silver (nano-Ag), selenium NP (nano-Se), and zinc oxide NP (nano-ZnO) are high production volume metal NPs that find application in a variety of industrial processes, manufacturing, agriculture, and nanomedicine. However, due to their small size, large surface area, and potential prooxidant capabilities, there are concerns regarding their toxicologic potential. 

Exposure of mice to nano-TiO_2_ has been shown to cause oxidative stress, gliosis, alteration in the expression of genes associated with memory and cognition, as well as the death of hippocampal neurons [222]. Inhalation of nano-TiO_2_ has been shown to augment BBB permeability and inflammatory cytokine production in the brain of aged rats [223]. Nano-Ag has been shown to upregulate the expression of inflammatory and antioxidant genes such as interleukin 1 (IL1), C-X-C motif chemokine 13 (CXCL13), macrophage receptor with collagenous structure (MARCO), and glutathione synthetase (GSS) [184,232]. Exposure to nano-CuO has been shown to decrease the spontaneous excitatory postsynaptic currents (sEPSCs) and miniature EPSCs (mEPSCs) and diminish pre-synaptic and post-synaptic glutamate neurotransmission, indicating reduced long-term potentiation (LTP) and cognitive dysfunction [233]. Exposure to nano-Se has been shown to cause enhanced Ca^2+^ signaling selectively within the astrocytes, increase lactate release, suppress hyperexcitation of neural networks, and activate A2-type astrocytes [234].

### 3.3. Gliosis in Neurological Disease States

Microglia play a critical role in regulating CNS physiology in the healthy brain. They are highly ramified structures that maintain direct contact with the dendritic spines, axons, and synapses of neurons, suggesting they are key participants in activity-dependent processes such as the regulation of the synaptic structure and function [235,236,237,238]. Like microglia, astrocytes also regulate brain signaling by modulating synapses, regulating homeostasis, transporting nutrients, and maintaining structural support [239,240].

#### 3.3.1. Gliosis in AD

**Figure 2:**
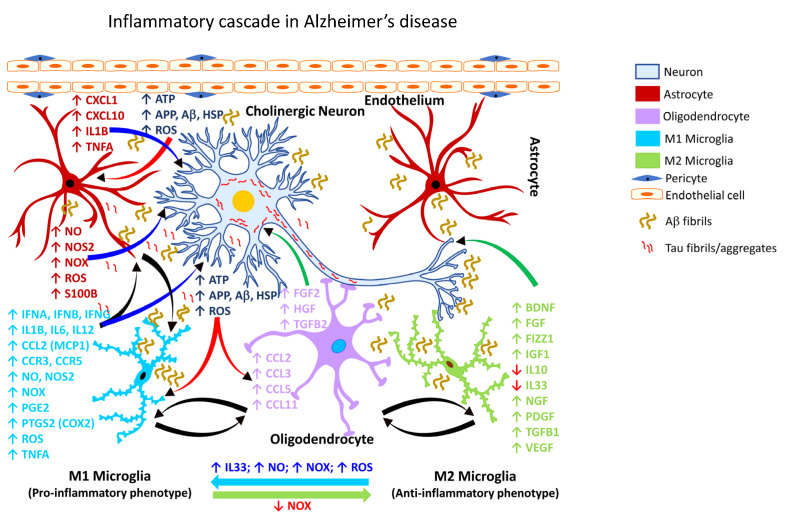
Inflammatory cascade in Alzheimer’s disease. Schematic representation of the molecular mechanisms associated with glia-mediated functional interactions and systematic perturbations within the CNS to induce neuroinflammation in Alzheimer’s disease. The endothelial cells form the inner lining of the blood vessel with pericytes enveloping the surface of the vasculature forming tight junctions to maintain the BBB integrity. Upon insult, the augmented production and release, as well as impaired clearance of Aβ and Tau fibrils sustains chronic activation of the primed microglia resulting in the production and release of proinflammatory mediators including free radicals, cytokines and chemokines, thereby affecting the resident CNS cells (astrocytes, oligodendrocytes, and neurons) and leading to Aβ and Tau aggregation. M1 microglia (proinflammatory phenotype, neurotoxic) release various proinflammatory mediators including free radicals, cytokines, and chemokines that further stimulate other glial cells and collectively contribute to exacerbating the neuronal injury/damage. M2 microglial cells (anti-inflammatory phenotype, neuroprotective) can polarize to an M1 state and release proinflammatory mediators in the presence of increased levels of NOX, ROS, NO, and IL33 released by M1 microglia and/or astrocytes and thereby augment the neuroinflammatory and neuronal injury process leading to synaptic dysfunction, neuronal injury, and neuronal death. Astrocytes respond by releasing proinflammatory mediators including free radicals, cytokines, and chemokines, which further contribute to enhancing the endothelial permeability, disrupting BBB integrity, and allowing for infiltration of peripheral immune cells, events that further intensify inflammation and neuronal injury. Feedback regulation of NOX or its inhibition causes M1 microglia to polarize to the M2 state (anti-inflammatory phenotype), which downregulates M1 functions and promotes regulation of neuroinflammation and neurorepair by releasing anti-inflammatory mediators such as cytokines, neurotrophic, and growth factors. Mediators released by specific neural cell types (neuron, astrocyte, microglia, or oligodendrocyte) are listed adjacent to each cell type in similar colored text. Curved arrows indicate the direction of signal flow between various neural cells for the inflammation activation process. Red curved arrows show the directional flow of danger/damage signals from neurons to glial cells (astroglia, microglia, oligodendroglia); blue curved arrows show the flow of proinflammatory signals from astrocytes and M1 microglia towards distressed neurons; green curved arrows show the flow of neurotrophic signals from M2 microglia and oligodendroglia towards the distressed neurons as a neuroprotective/neurorescue endeavor; black curved arrows show the directional crosstalk among various glial cells to mount a glial response to neuronal injury/damage. ↑, increase; ↓, decrease; Aβ, beta amyloid; APP, amyloid precursor protein; ATP, adenosine triphosphate; BBB, blood-brain barrier; BDNF, brain-derived neurotrophic factor; CCL2, C-C motif chemokine ligand 2 (also referred to as MCP1, monocyte chemoattractant protein 1); CCL3, C-C motif chemokine ligand 3; CCL5, C-C motif chemokine ligand 5; CCL11, C-C motif chemokine ligand 11; CCR3, C-C motif chemokine receptor 3; CCR5, C-C motif chemokine receptor 5; 1; CXCL1, C-X-C motif chemokine ligand 1; CXCL10, C-X-C motif chemokine ligand 10; FGF, fibroblast growth factor; FGF2, fibroblast growth factor 2; FIZZ1, found in inflammatory zone 1; HGF, hepatocyte growth factor; HSP, heat shock proteins; IFNA, interferon alpha; IFNB, interferon beta; IFNG, interferon gamma; IGF1, insulin-like growth factor 1; IL1B, interleukin 1 beta; IL6, interleukin 6; IL10, interleukin 10; IL12, interleukin 12; IL33, interleukin 33; NGF, nerve growth factor; NO, nitric oxide; NOS2, nitric oxide synthase 2 (inducible nitric oxide synthase); NOX, NADPH oxidase 1; PDGF, platelet-derived growth factor; PGE2, prostaglandin E2; PTGS2, prostaglandin-endoperoxide synthase 2 (also referred to as COX2, cyclooxygenase 2); ROS, reactive oxygen species; S100B calcium binding protein B; TGFB1, transforming growth factor beta 1; TGFB2, transforming growth factor beta 2; TNFA, tumor necrosis factor alpha; VEGF, vascular endothelial growth factor.

It is estimated that by the year 2050, more than 150 million people worldwide will be impacted by some form of dementia, with AD accounting for nearly 70% of such dementia cases [240,241]. Chronic neuroinflammation is a prominent feature of AD pathology [242] manifested by reactive gliosis and robust expression of proinflammatory mediators, associated with synapse loss [243,244]. Aβ causes functional and morphological changes in the neighboring astrocytes, eliciting an astroglial response (Figure 2; Table 3). 

Aβ-mediated microglial activation (Figure 2; Table 3) can also downregulate homeostatic genes, such as C-X-3-C motif chemokine receptor (*CX3CR1), P2RY12*, and genes involved in cell adhesion, lipid signaling, and G protein-coupled receptor (GPCR) pathways [273,274]. Complement factors have also been implicated in the synaptic neurotoxicity and neurodegeneration associated with AD. For example, pharmacological inhibition of the complement pathway has been shown to ameliorate synapse loss and neurodegeneration in murine models of AD [244,275].

Recently, a bidirectional interaction of the nervous and immune systems has been demonstrated [276]. The study showed that a systemic inflammatory event could elicit selective neuronal activation within the insular lobe of the cortex, and a subsequent insult of this immune-imprinted neuronal population recapitulated the primary inflammatory episode in the peripheral target [276]. It is also well-recognized that the breakdown of BBB facilitates the infiltration of toxicants and immune cells into the brain, causing neuroinflammation and subsequent activation of downstream cascades associated with neural injury and neurodegeneration [277]. 

Post-mortem analysis of brains from patients with AD has revealed abnormal accumulation of metal ions such as Fe, copper (Cu), and zinc (Zn), suggesting a role for dysregulated redox metals in the pathogenesis of AD. Specifically, deposits of metals, e.g., Fe, Cu, and Zn have been found in the rim and core of senile plaques and appear to co-localize with Aβ aggregates [278,279]. Fe causes lipid peroxidation via iron-dependent oxidases such as lipoxygenase, which activates ferroptosis and AD [280]. Zn in the synaptic cleft has been shown to be neurotoxic because it inhibits NMDAR and increases α-amino-3-hydroxy-5-methyl-4-isoxazole propionic acid receptor (AMPAR)-mediated toxicity [281], thus affecting memory regulation in AD [282]. Zn is also known to interact with Aβ and aggravate pathogenesis due to Zn dyshomeostasis in AD [282]. 

Among the several proposed mechanisms of neuroinflammation in AD, a recent study has shown that the soluble epoxide hydroxylase is aberrantly elevated in the brains of patients with AD, and in transgenic or knock-in mouse models of AD [283]. Specifically, increased soluble epoxide hydrolase levels were reported to occur within the astrocytes. Functionally, the soluble epoxide hydrolase is known to rapidly scavenge the anti-inflammatory arachidonic acid derivatives, thereby facilitating the progression of the inflammatory process [283].

#### 3.3.2. Gliosis in PD

PD is a chronic and progressive neurodegenerative disease characterized by motor and non-motor dysfunction, with the former being attributable to the loss of nigrostriatal dopaminergic neurons. The clinical motor dysfunction phenotype comprises of a complex of symptoms including muscle rigidity, resting tremor bradykinesia, and postural instability, collectively termed parkinsonism. The non-motor dysfunction typically involves neurons outside of the dopaminergic pathway, and the clinical phenotype is characterized by sleep disruption, cognitive impairment, and depression [284,285]. Numerous transgenic animal models have been exploited to study PD, including *SNCA (PARK1)*, parkin RBR E3 ubiquitin protein ligase (PRKN/PARK2/Parkin), PTEN-induced kinase 1 (*PINK1/PARK6*), parkinsonism-associated deglycase (*DJ1/PARK7*), and leucine-rich repeat kinase 2 (*LRRK2/PARK8*). These models capture many of the salient features of PD, but none solely recapitulate all the cardinal features of dopaminergic neurodegeneration [286]. For example, while exogenous αSYN (SNCA) leads to mitochondrial dysfunction, the overexpression of PRKN rescues mitochondrial dysfunction [287]. The degeneration of the nigrostriatal pathway is always associated with extra-nigral dysfunction involving the dorsal motor nucleus of the glossopharyngeal and vagal nerves, thalamic sub-nuclei, amygdala, and the neocortex [274,288]. The pathology in PD evolves in stages, beginning with lesions of the dorsal IX/X motor nuclei in the medulla oblongata (stage 1), progressing to the gigantocellular reticular nucleus and caudal raphe nuclei in the medulla oblongata and pontine tegmentum (stage 2), substantia nigra pars compacta/SNpc in the midbrain (stage 3), and the cortical areas, mesocortex (stage 4) and neocortex (stages 5 and 6) [289]. Further, the non-motor symptoms often precede motor dysfunction by several years or decades [289]. Experimental neurotoxic models of PD also include the use of chemical agents, e.g., 1-methyl-4-phenyl-1,2,3,6-tetrahydropyridine (MPTP), 6-hydroxydopamine (6-OHDA), as well as pesticides and herbicides, e.g., rotenone and paraquat [290].

Multiple lines of investigations have shown that reactive microgliosis is associated with PD (Table 4). 

Reactive microgliosis occurs in the midbrain, striatum (primarily the caudate and putamen), hippocampal formation, and cortical regions of post-mortem brains obtained from patients with PD [313], following recreational administration of the dopaminergic neurotoxicant MPTP [314], or in experimental models of PD using MPTP [315,316,317,318]. Microglial inflammation-related activation of the aldosterone and metabolic pathways generating ROS also appears to be altered in PD, besides synaptic activity, neurotransmission, and neuronal injury or rescue [319].

**Figure 3:**
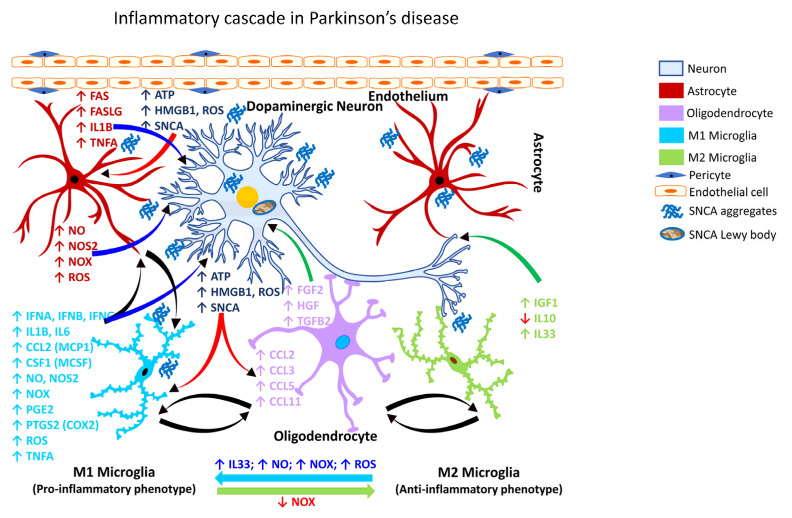
Inflammatory cascade in Parkinson’s disease. Schematic representation of the molecular mechanisms associated with glia-mediated functional interactions and systematic perturbations within the CNS to induce neuroinflammation in Parkinson’s disease. The endothelial cells form the inner lining of the blood vessel with pericytes enveloping the surface of the vasculature forming tight junctions to maintain the BBB integrity. Upon insult, the augmented production and release, as well as impaired clearance of αSYN (SNCA) sustains chronic activation of the primed microglia resulting in the production and release of proinflammatory mediators including free radicals, cytokines and chemokines, thereby affecting the resident CNS cells (astrocytes, oligodendrocytes, and neurons) and leading to αSYN (SNCA) aggregation, neuronal injury and Lewy body formation. M1 microglia (proinflammatory phenotype, neurotoxic) release various proinflammatory mediators, including free radicals, cytokines, and chemokines that further stimulate other glial cells and collectively contribute to exacerbating neuronal injury/damage. M2 microglial cells (anti-inflammatory phenotype, neuroprotective) can polarize to an M1 state and release proinflammatory mediators in the presence of increased levels of NOX, ROS, NO, and IL33 released by M1 microglia and/or astrocytes and thereby augment the neuroinflammatory and neuronal injury process leading to synaptic dysfunction, neuronal injury, and neuronal death. Astrocytes respond by releasing proinflammatory mediators, including free radicals, cytokines, and chemokines, which further contribute to enhancing endothelial permeability, disrupting BBB integrity, and allowing for infiltration of peripheral immune cells, events that further intensify inflammation and neuronal injury. Feedback regulation of NOX or its inhibition causes M1 microglia to polarize to the M2 state (anti-inflammatory phenotype), which downregulates M1 functions and promotes regulation of neuroinflammation and neurorepair by releasing anti-inflammatory mediators such as cytokines, neurotrophic, and growth factors. Mediators released by specific neural cell types (neuron, astrocyte, microglia, or oligodendrocyte) are listed adjacent to each cell type in similar colored text. Curved arrows indicate the direction of signal flow between various neural cells for the inflammation activation process. Red curved arrows show the directional flow of danger/damage signals from neurons to glial cells (astroglia, microglia, oligodendroglia); blue curved arrows show the flow of proinflammatory signals from astrocytes and M1 microglia towards distressed neurons; green curved arrows show the flow of neurotrophic signals from M2 microglia and oligodendroglia towards the distressed neurons as a neuroprotective/neurorescue endeavor; black curved arrows show the directional crosstalk among various glial cells to mount a glial response to neuronal injury/damage. ↑, increase; ↓, decrease; ATP, adenosine triphosphate; BBB, blood-brain barrier; CCL2, C-C motif chemokine ligand 2 (also referred to as MCP1, monocyte chemoattractant protein 1); CCL3, C-C motif chemokine ligand 3; CCL5, C-C motif chemokine ligand 5; CCL11, C-C motif chemokine ligand 11; CSF1, colony-stimulating factor 1; FAS, Fas cell surface death receptor; FASLG, Fas ligand; FGF2, fibroblast growth factor 2; HGF, hepatocyte growth factor; HMGB1, high-mobility group box 1; IFNA, interferon alpha; IFNB, interferon beta; IFNG, interferon gamma; IGF1, insulin-like growth factor 1; IL1B, interleukin 1 beta; IL6, interleukin 6; IL10, interleukin 10; IL33, interleukin 33;NO, nitric oxide; NOS2, nitric oxide synthase 2 (inducible nitric oxide synthase); NOX, NADPH oxidase 1; PGE2, prostaglandin E2; PTGS2, prostaglandin-endoperoxide synthase 2 (also referred to as COX2, cyclooxygenase 2); ROS, reactive oxygen species; αSYN/SNCA, alpha synuclein; TGFB2, transforming growth factor beta 2; TNFA, tumor necrosis factor alpha.

Recently, it has been shown that αSYN (SNCA) can promote neurotoxic astrocyte activation (Figure 3; Table 4), and receptor-interacting serine/threonine kinase 1 (RIPK1; also known as receptor-interacting protein/RIP) signaling can regulate glial cell biology and neuroinflammation [320]. The role of αSYN (SNCA) in astrocyte activation was previously unknown until it was shown that that pre-formed αSYN (SNCA) fibrils can induce A1 and A2 activation states in astrocytes isolated from the human midbrain [320].

#### 3.3.3. Gliosis in CJD

Creutzfeldt-Jackob disease (CJD) is characterized as a rapidly progressive, fatal, transmissible neurodegenerative disease linked to the accumulation of untreatable prion protein (PrP^c^) resulting in encephalopathy and neurodegenerative disorders in the CNS [321,322]. Early diagnosis of CJD remains a major clinical challenge because the manifestation of prion disease at onset are inconsistent and often nonspecific. CJD affects humans and many other mammalian species [237,323]. 

Broadly, CJD is categorized into three subtypes: sporadic, inherited, and acquired. The most interesting characteristics of sporadic CJD are pathological features implicated during the development of spongiform change in the cerebral gray matter, which is further associated with the deposition of abnormal forms of prion protein (PrP^sc^), resulting in cognitive and motor dysfunction [324]. While the etiology of sporadic CJD remains unknown, it is hypothesized that a somatic mutation in the prion protein (PRNP) or misfolding of PrP^c^ into PrP^sc^ [325] might underlie the etiopathogenesis of CJD. Rapidly progressive dementia is typical in CJD, and the patients generally die within one year after clinical onset [326].

Aside from the prion protein-related hypothesis, it is suggested that the cytokine profile in CJD can be pro-inflammatory and anti-inflammatory, with increases seen in the inflammatory cytokine interleukin 8 (IL8) and a decrease in transforming growth factor beta 2 (TGFB2) but no changes in the levels of interleukin 1 beta (IL1B), interleukin 12 (IL12), or tumor necrosis factor alpha (TNFA) [327]. The regulatory protein PU1, interleukin 34 (IL34), and CCAAT enhancer binding protein alpha (CEBPA) is also involved in microglial proliferation in CJD [237,328]. Additionally, prostaglandin-endoperoxide synthase 1 (PTGS1/COX1), prostaglandin-endoperoxide synthase 2 (PTGS2/COX2), and prostaglandin E2 (PGE2) have been reported to be elevated in CJD [329,330], supporting the involvement of inflammation. Further, it has been shown that the increased inflammatory response seen in CJD is associated with the activation of NFκB and STAT3 signaling pathways [330]. Numerous studies have also reported signs of oxidative stress [134,237,331,332], and activated microglia in the brains of patients with CJD [333]. 

#### 3.3.4. Gliosis in ALS

Amyotrophic lateral sclerosis (also known as Lou Gehrig’s disease) is a progressive neurodegenerative disorder affecting the motor neurons in the cerebral cortex, brainstem, and spinal cord. While the underlying mechanism of the neuronal degeneration remains elusive, a pathological basis involving the ubiquitin-immunoreactive cytoplasmic inclusions has been suggested, which is associated with a robust inflammatory reaction [334]. In ALS, mutations in the TAR DNA binding protein (TARDBP) are usually rare despite ALS patients exhibiting cytoplasmic aggregates of TDP43 in the affected brain areas [335].

Recent studies have shown that a deficiency of the guanine nucleotide exchange factor C9orf72 (C9orf72) alters the homeostatic gene signature of microglia [336] and contributes to the loss of synaptic processes [336]. Another line of investigation demonstrated that pharmacological inhibition of the purinergic receptor P2X 7 (P2RX7) decreases microgliosis, inhibits the expression of NF-κB, and attenuates motor neuron death [274,337]. Further, studies of the spatiotemporal dynamics of microglial activation in SOD1G93A mice have shown that microglial dysfunction precedes the onset of the disease [338].

**Figure 4:**
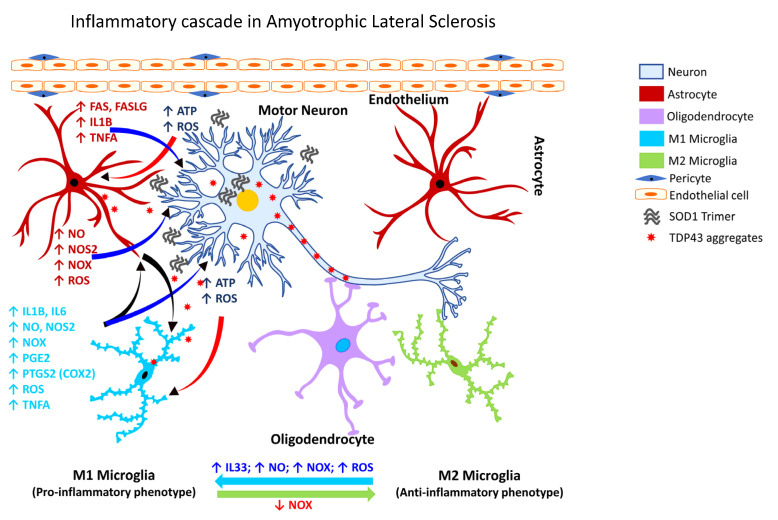
Inflammatory cascade in Amyotrophic Lateral Sclerosis. Schematic representation of the molecular mechanisms associated with glia-mediated functional interactions and systematic perturbations within the CNS to induce neuroinflammation in Parkinson’s disease. The endothelial cells form the inner lining of the blood vessel with pericytes enveloping the surface of the vasculature forming tight junctions to maintain the BBB integrity. Upon insult, the augmented production and release, as well as impaired clearance of mutant SOD1 trimers, TDP43, and ubiquitin aggregates sustains chronic activation of the primed microglia resulting in the production and release of proinflammatory mediators, including free radicals, cytokines, and chemokines, thereby affecting the resident CNS cells (astrocytes, oligodendrocytes, and neurons) and leading to disruption of nuclear-cytoplasmic transport, ubiquitination and accumulation of TDP43 in the cytoplasm, aggregation of SODI trimers, and subsequent neuronal injury/damage. M1 microglia (proinflammatory phenotype, neurotoxic) release various proinflammatory mediators including free radicals, cytokines and chemokines that further stimulate other glial cells and collectively contribute to exacerbating the neuronal injury/damage. M2 microglial cells (anti-inflammatory phenotype, neuroprotective) can polarize to an M1 state and release proinflammatory mediators in the presence of increased levels of NOX, ROS, NO, and IL33 released by M1 microglia and/or astrocytes and thereby augment the neuroinflammatory and neuronal injury process leading to synaptic dysfunction, neuronal injury, and neuronal death. Astrocytes respond by releasing proinflammatory mediators, including free radicals, cytokines, and chemokines, which further contribute to enhancing the endothelial permeability, disrupting BBB integrity, and allowing for infiltration of peripheral immune cells, events that further intensify inflammation and neuronal injury. Feedback regulation of NOX or its inhibition causes M1 microglia to polarize to the M2 state (anti-inflammatory phenotype), which downregulates M1 functions and promotes regulation of neuroinflammation and neurorepair by releasing anti-inflammatory mediators, e.g., cytokines, neurotrophic, and growth factors. Mediators released by specific neural cell types (neuron, astrocyte, microglia or oligodendrocyte) are listed adjacent to each cell type in similar colored text. Curved arrows indicate the direction of signal flow between various neural cells for the inflammation activation process. Red curved arrows show the directional flow of danger/damage signals from neurons to glial cells (astroglia, microglia, oligodendroglia); blue curved arrows show the flow of proinflammatory signals from astrocytes and M1 microglia towards distressed neurons; green curved arrows show the flow of neurotrophic signals from M2 microglia and oligodendroglia towards the distressed neurons as a neuroprotective/neurorescue endeavor; black curved arrows show the directional crosstalk among various glial cells to mount a glial response to neuronal injury/damage. ↑, increase; ↓, decrease; ATP, adenosine triphosphate; BBB, blood-brain barrier; FAS, Fas cell surface death receptor; FASLG, Fas ligand; IL1B, interleukin 1 beta; IL6, interleukin 6; IL33, interleukin 33; NO, nitric oxide; NOS2, nitric oxide synthase 2 (inducible nitric oxide synthase); NOX, NADPH oxidase 1; PGE2, prostaglandin E2; PTGS2, prostaglandin-endoperoxide synthase 2 (also referred to as COX2, cyclooxygenase 2); ROS, reactive oxygen species; SOD1, superoxide dismutase 1; TNFA, tumor necrosis factor alpha.

Cumulative evidence over the past two decades shows microglial activation is associated with the degeneration seen in patients with ALS, as assessed by PET analysis. One of the earliest studies revealed diffuse microglial activation in both the motor and non-motor regions of the cerebral cortex [274,335,339,340,341,342]. Additionally, activation of glia in ALS (Figure 4) is associated with marked elevation of ROS, and inflammatory mediators, e.g., PTGS2 (COX2), IL1B, interleukin 6 (IL6), and TNFA [334].

#### 3.3.5. Gliosis in MS

MS is a heterogeneous autoimmune, and complex inflammatory disease of the CNS characterized by demyelinating lesions. The inflammatory outcome in MS is attributed to microglia, however, the extent of their involvement in the disease process and the underlying molecular mechanisms through the mediation of neural injury or damage is still unclear [343]. Despite this knowledge gap, the presence of activated microglia at the sites of MS lesions seems to be a common feature. Patients with a progressive course of MS exhibit axonal degeneration and chronic active lesions with microglia typically present at the rim of the pathological lesions [274,344] in association with complement factors, antibodies, and immune cells [345,346]. Phagocytic microglia have also been observed in the white matter of the brain tissues obtained from patients with secondary progressive MS [347].

**Figure 5:**
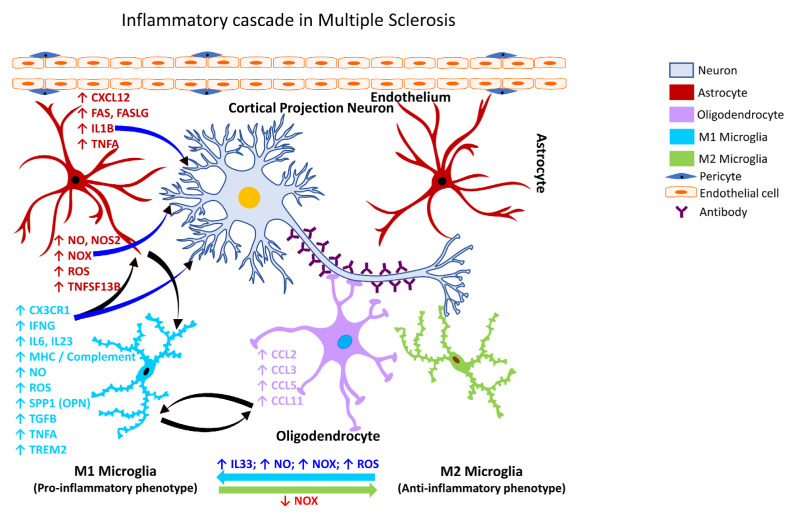
Inflammatory cascade in Multiple Sclerosis. Schematic representation of the molecular mechanisms associated with glia-mediated functional interactions and systematic perturbations within the CNS to induce neuroinflammation in Multiple Sclerosis. The multifocal inflammatory demyelination of the white matter is driven largely by an inflammatory process besides an autoimmune component that involves innate and adaptive (B and T lymphocytes) immune cells. Various subtypes of myeloid cells are also critical for the pathogenic implications and the blood-derived monocytes represent the highest fraction of infiltrating peripheral cells into the CNS that undergo transformation into monocyte-derived inflammatory phagocytes (macrophages or dendritic cells) leading to neuronal damage. The endothelial cells form the inner lining of the blood vessel with pericytes enveloping the surface of the vasculature forming tight junctions to maintain the BBB integrity. Disruption of the BBB integrity further facilitates the infiltration of autoreactive immune cells into the CNS. Microglia serve as antigen presenting cells and present the myelin antigen to infiltrating B and T-cells, thereby exacerbating the neuroinflammatory cascade. M1 microglia (proinflammatory phenotype, neurotoxic) release various proinflammatory mediators, including free radicals, cytokines, and chemokines that further stimulate other glial cells, thus perpetuating a self-destructive environment that collectively contributes to exacerbating the neuronal injury/damage. M2 microglial cells (anti-inflammatory phenotype, neuroprotective) can polarize to an M1 state and release proinflammatory mediators in the presence of increased levels of NOX, ROS, NO, and IL33 released by M1 microglia and/or astrocytes and thereby augment the neuroinflammatory and neuronal injury process leading to synaptic dysfunction, neuronal injury, and neuronal death. Astrocytes respond by releasing proinflammatory mediators, including free radicals, cytokines, and chemokines, which further contribute to enhancing the endothelial permeability, disrupting BBB integrity, and allowing for infiltration of peripheral immune cells, events that further intensify inflammation and neuronal injury. Feedback regulation of NOX or its inhibition causes M1 microglia to polarize to the M2 state (anti-inflammatory phenotype), which downregulates M1 functions and promotes regulation of neuroinflammation and neurorepair by releasing anti-inflammatory mediators, e.g., cytokines, neurotrophic, and growth factors. Mediators released by specific neural cell types (neuron, astrocyte, microglia or oligodendrocyte) are listed adjacent to each cell type in similar colored text. Curved arrows indicate the direction of signal flow between various neural cells for the inflammation activation process. Red curved arrows show the directional flow of danger/damage signals from neurons to glial cells (astroglia, microglia, oligodendroglia); blue curved arrows show the flow of proinflammatory signals from astrocytes and M1 microglia towards distressed neurons; green curved arrows show the flow of neurotrophic signals from M2 microglia and oligodendroglia towards the distressed neurons as a neuroprotective/neurorescue endeavor; black curved arrows show the directional crosstalk among various glial cells to mount a glial response to neuronal injury/damage. ↑, increase; ↓, decrease; BBB, blood-brain barrier; CCL2, C-C motif chemokine ligand 2 (also referred to as MCP1, monocyte chemoattractant protein 1); CCL3, C-C motif chemokine ligand 3; CCL5, C-C motif chemokine ligand 5; CCL11, C-C motif chemokine ligand 11; CXCL12, C-X-C motif chemokine ligand 12; CX3CR1, C-X3-C motif chemokine receptor 1 (also referred to as fractalkine receptor); FAS, Fas cell surface death receptor; FASLG, Fas ligand; IFNG, interferon gamma; IL1B, interleukin 1 beta; IL6, interleukin 6; IL23, interleukin 23; IL33, interleukin 33; MHC, major histocompatibility complex; NO, nitric oxide; NOS2, nitric oxide synthase 2 (inducible nitric oxide synthase); NOX, NADPH oxidase 1; ROS, reactive oxygen species; SPP1, secreted phosphoprotein 1; TGFB, transforming growth factor beta; TNFA, tumor necrosis factor alpha; TNFSF13B, TNF superfamily member 13b; TREM2, triggering receptor expressed on myeloid cells 2.

Following activation of NADPH Oxidase 2 (CYBB/NOX2), microglia increase their chemotactic signaling and recruit peripheral immune cells to the brain (Figure 5), which play a role in demyelination and axonal damage [348]. The intersection of MS pathogenesis and autoreactive T and B lymphocytes has been gaining interest in recent years as they appear to play a role as amplifiers and effectors in MS. Specifically, a subset of T-helper cells, the Th17 cells, appear to play a role in the pathogenesis of MS [349] through the secretion of IL12 and related members, interleukin 21 (IL21), and interleukin 23 (IL23). Further, IL21 is known to induce the activation of Th17 cells in an autocrine manner [350]. Other factors involved in the inflammatory pathogenesis of MS include polymorphisms in the T-cell receptor beta locus (TRB), cytotoxic T-lymphocyte associated protein 4 (CTLA4), CD6 molecule (CD6), interleukin 10 (IL10), interleukin 2 receptor subunit beta (IL2R), interleukin 4 receptor (IL4R), interleukin 7 receptor (IL7R), C-C motif chemokine receptor 5 (CCR5), interferon-gamma (IFNG), interferon regulatory factor 8 (IRF8), intercellular adhesion molecule 1 (ICAM1), TNFA, TNF receptor superfamily member 1A (TNFRSF1A/TNFRI, vitamin D receptor (VDR), and estrogen receptors 1 and 2 (ESR1 and ESR2) [351].

## 4. Signaling Pathways Associated with Reactive Gliosis

### 4.1. Protein Phosphorylation in Neural Injury and Gliosis

One of the significant interests in astroglial biology is to know how the astrocytes, critical for synapse formation and neuronal maintenance, transform into a pathogenic form. As protein phosphorylation is a principal regulatory mechanism controlling almost all cellular machinery, the generalization is that it may actively modulate astrocyte and microglial communication and response. Despite the significant role of astrocytes in neurodegeneration, the cellular and molecular mechanisms that promote pathogenic astrocyte activity remain unresolved [320].

In the eukaryotic cell, protein phosphorylation refers to the reversible, covalent addition of a phosphate group to the serine, threonine, or tyrosine side chains of acceptor amino acid residues mediated by protein kinases [352,353]. Further, phosphorylation of protein kinases themselves can be associated with the injury process. For example, phosphorylation of the protein kinase R-like endoplasmic reticulum kinase (PERK) generates a distinct reactive state in astrocytes, which alters the astrocytic secretome leading to loss of synaptic function [354]. In addition, phosphorylation of PERK at specific residues has been shown to alter the function of proteins electrostatically by modulating the intermolecular interactions or by allosterically modifying the protein conformation, eventually influencing the protein activity [355].

Proliferation and apoptosis adaptor protein 15 (PEA15), a phosphoprotein enriched in astrocytes, is a cytoplasmic protein that regulates cell proliferation and apoptosis [356]. It has been shown to act as a cytoplasmic anchor for mitogen-activated protein kinase 3 and 1 (MAPK3/1; also known as ERK1/2) and prevents it from translocating to the nucleus, thus interfering with or reducing ERK1/2-mediated gene expression [357]. PEA15 protein is essential for the normal functioning of mature astrocytes [358]. Its levels and phosphorylation state exhibit peak expression in the adult brain [359]. Biogenic neurotransmitters such as noradrenaline, and hormones such as endothelin and vasoactive intestinal peptide are known to modulate the phosphorylation of PEA15 through the activation of kinases, primarily protein kinase C and calcium-calmodulin-dependent protein kinase II, thereby enhancing the intracellular levels of Ca^2+^ in the astrocytes [358]. Increased Ca^2+^ is known to elicit reactive gliosis and increase GFAP immunoreactivity, which is thought to be mediated by the activation of calpain I [360].

### 4.2. Activation of NF-κB Pathway in Neural Injury and Gliosis

**Figure 6:**
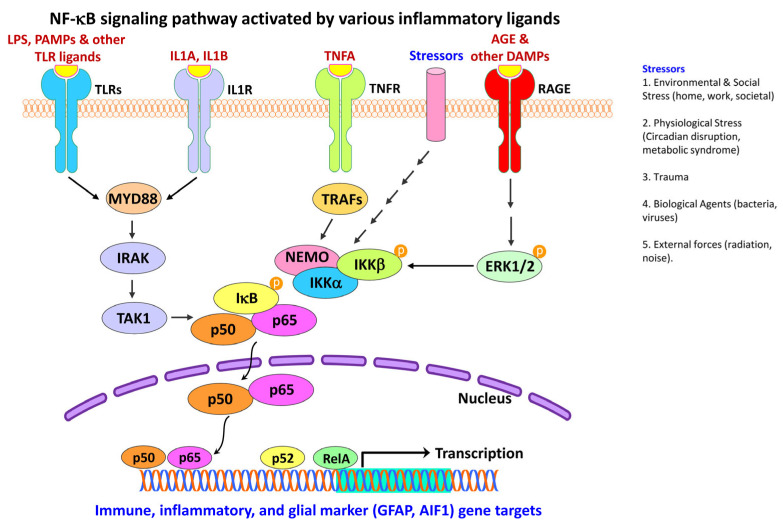
NF-κB signaling in neuroinflammatory response. Schematic representation depicts activation and regulation of NF-κB signaling pathways by various inflammatory ligands. NF-κB activation is initiated when ligands stimulate various cell-surface receptors, e.g., TLR2, IL1R, TNFR or RAGE. Upon stimulation, TLRs and IL1R assembles the cytoplasmic MYD88 complex, which recruits IRAK and TAK1. Stimulation of TNFR recruits TRAFs. TAK1 and TRAFs activate the downstream kinase IKK, which in turn phosphorylate the NF-κB inhibitor IκBα, leading to ubiquitin-dependent IκBα degradation and NF-κB activation. Once activated, TRAFs function as an E3 ubiquitin ligase that catalyzes the synthesis of K63-linked polyubiquitin chains conjugated to itself, NEMO. A complex signal transduction process then starts as soon as TNFRs are activated. IKK is ultimately triggered and leads to the phosphorylation of IκB, which results in IκB ubiquitination and degradation. When IκB is degraded, the remaining NF-κB dimer (e.g., p65/p50 or p50/p50 subunit or their putative combination) translocates to the nucleus, which further binds to a DNA consensus sequence of target immune, inflammatory or glial marker (e.g., GFAP, AIF1) genes. Similarly, the binding of AGE and other DAMP ligands to RAGE receptor, causes ERK1/2 phosphorylation that further leads to activation and phosphorylation of the NEMO-IKK complex, NF-κB activation, and subsequent translocation of the NF-κB dimer (e.g., p65/p50 or p50/p50 subunit or their putative combination) to the nucleus to regulate transcription of immune and inflammatory genes including glial markers, e.g., GFAP, AIF1. Over-expression of RAGE produces vicious cycles that perpetuate oxidative stress and contribute to neuroinflammation by nuclear factor-kB (NF-kB) up-regulation. Black arrows indicate the activation process and multiple arrows indicate several unknown intermediates in the signaling cascade. AGE, advanced glycation end-products; AIF1, allograft inflammatory factor 1 (also known as IBA1, ionized calcium-binding adapter molecule 1); DAMP, damage-associated molecular patterns; ERK1/2, extracellular signal-regulated kinase 1 and 2; GFAP, glial fibrillary acidic protein; IKKα, inhibitor of nuclear factor kappa-B kinase subunit alpha (IKKA; also known as CHUK, component of inhibitor of nuclear factor kappa B kinase complex); IKKβ, inhibitor of nuclear factor kappa-B kinase subunit beta (IKKB; also known as IKBKB); IL1A, interleukin 1 alpha; IL1B, interleukin 1 beta; IL1R, interleukin 1 receptor; IRAK, interleukin 1 receptor associated kinase 1; IRAK, interleukin 1 receptor-associated kinase; IκB, inhibitor of nuclear factor kappa B; LPS, lipopolysaccharide; MYD88, MYD88 innate immune signal transduction adaptor (also known as myeloid differentiation primary response gene 88; NEMO, NF-kappaB essential modulator (also known as IKBKG, inhibitor of nuclear factor kappa-B kinase regulatory subunit gamma); NF-κB, nuclear factor kappa B; P, phosphate group; p50, protein p50 (the functional subunit of the 105 kD precursor protein coded by the gene NFKB1, nuclear factor kappa B subunit 1); p52, protein p52 (the functional subunit of the 100 kD precursor protein coded by the gene NFKB2, nuclear factor kappa B subunit 2); p65, protein p65 (also known as RELA, RELA proto-oncogene, NF-KB Subunit or V-Rel avian reticuloendotheliosis viral oncogene homolog A); PAMPs, pathogen-associated molecular patterns; RAGE, receptor for advanced glycation end-products; TAK1, transforming growth factor beta activated kinase 1; TLR, toll-like receptor; TNFA, tumor necrosis factor alpha; TNFR, tumor necrosis factor receptor; TRAF, tumor necrosis actor receptor-associated factor.

NF-κB is a family of pleiotropic inducible transcription factors discovered in 1986 that are well known to regulate both innate and adaptive immune functions, besides serving as a pivotal signaling pathway (Figure 6) for inflammatory stimuli [361,362]. NF-κB signaling has been categorized into two major pathways, the canonical and non-canonical pathways, both central to the regulation of the immune and inflammatory response despite their distinct signaling mechanisms [362,363].

The NF-κB family consists of five related structural members: nuclear factor kappa B subunit 1 (NFKB1; p50), nuclear factor kappa B subunit 2 (NFKB2; p52), REL proto-oncogene, NF-KB subunit (REL; also known as V-Rel avian reticuloendotheliosis viral oncogene homolog; c-Rel), RELA proto-oncogene, NF-KB subunit (RELA/NFKB3; also known as V-Rel avian reticuloendotheliosis viral oncogene homolog A; p65), and RELB proto-oncogene, NF-KB subunit (RELB; also known as V-Rel avian reticuloendotheliosis viral oncogene homolog B). These five members mediate the transcription of target genes by binding to a specific DNA element, the κB enhancer, through a variety of homo- or hetero-dimer formations [4,362]. Deregulation of NF-κB is a hallmark of chronic inflammatory diseases [362].

Within the nucleus, active NF-κB promotes the transcription of NF-κB-dependent genes, such as the NLR family pyrin domain containing 3 (NLRP3), interleukin 1 beta (IL1B), and interleukin 18 (IL18), key components of the inflammasome [362]. NF-κB also has a role in regulating the activation of inflammasomes [364].

The activated NF-κB pathway is evident in the neurons of the hippocampus and entorhinal cortex of post-mortem brain samples from patients with AD [365,366], in the dopaminergic neurons within the SNPc of post-mortem brain samples from patients with PD [367], and in peripheral immune cells of patients with HD [368]. Increased accumulation of NF-κB in astrocytes is also reported in the R6/2 animal model of HD [369]. Inducible NF-κB has been detected at presynaptic sites in the cortex, hippocampus, and cerebellum and is known to be responsive to the agents such as glutamic acid (glutamate). Such NF-κB amounts are highly variable in the neuronal nucleus and are referred to as active NF-κB, as they are induced by neuronal synaptic activity [370].

NF-κB is also a core transcription factor for various inflammatory and immune mediators [371]. Following spinal cord injury, downregulation of SKI proto-oncogene (SKI) has been shown to inhibit glial inflammation by inhibiting the activity of the NF-κB, thus highlighting the role of NF-κB in the activation of astrocytes and the release of inflammatory mediators [372]. Other studies have shown that systemic inflammation or aging modulates hypothalamic function through NF-κB activation and microglia-neuron cross-talk [373,374]. Thus, NF-κB is a family of transcription factors that are strongly implicated in the physiological and pathological processes involving inflammation, immunity, cell proliferation, cell differentiation, and cell survival.

Inhibition of NF-κB in microglia was shown to reduce propagation and neurotoxicity of the human microtubule-associated protein tau, as well as restore learning and memory function in aged P301S transgenic mice (PS19 mice), which express the P301S mutant form [375]. NF-κB activation has also been shown to occur in the microglia of the SOD1^G93A^ mouse model of ALS, especially at the later stages of the disease [376]. Further, the reduction of the inhibitor of nuclear factor-kappa B kinase subunit beta (IKKB) levels and thus of NF-κB activity in the SOD1^G93A^ transgenic mice extended their median survival rate [274,376].

NF-κB mediates the synergistic activation of the positive-feedback loop for IL6-signaling (IL6 amplifier), which is further activated following IL6 and/or IL17 stimulation of non-immune cells [131]. IL6 amplifier is also activated by simultaneous stimulation of NF-κB and STAT3 to locally induce chemokines, causing chronic inflammation [131]. This provides evidence of crosstalk between the NF-κB and the JAK/STAT signaling pathways.

### 4.3. Activation of JAK/STAT Pathway in Neural Injury and Gliosis

**Figure 7:**
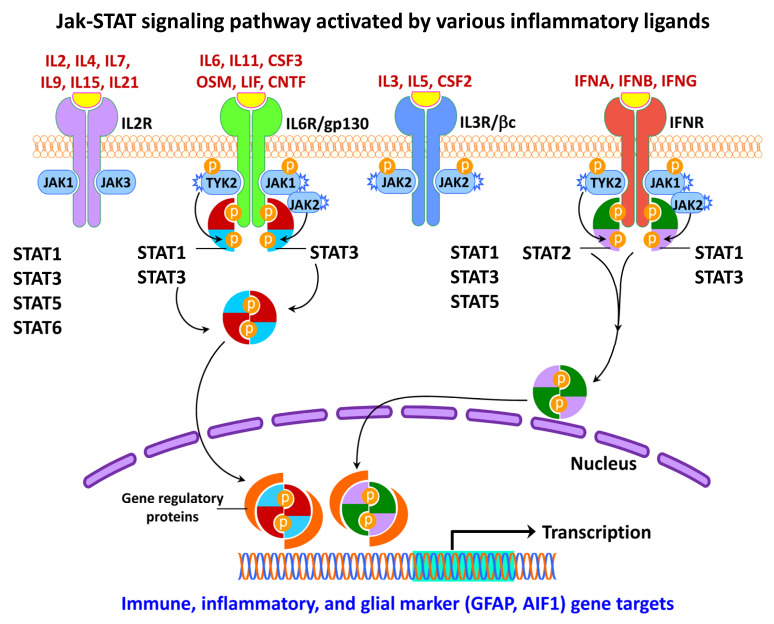
JAK-STAT signaling in neuroinflammatory response. Schematic representation depicting activation and regulation of JAK/STAT signaling pathways by various inflammatory ligands. The JAK family has four main members: JAK1, JAK2, JAK3, and TYK2. Black arrows indicate the activation process. Inflammatory mediators and growth factors bind to their corresponding receptors leading to receptor dimerization and recruitment of related JAKs. These mediators include, but are not limited to, interferons, such as interferon alpha, interferon beta, and interferon gamma; interleukin 2, interleukin 3, interleukin 4, interleukin 5, interleukin 6, interleukin 7, interleukin 9, interleukin 11, interleukin 15, interleukin 21; colony stimulating factor 2, colony stimulating factor 3; oncostatin M; leukemia inhibitory factor; and ciliary neurotrophic factor. JAK activation leads to tyrosine phosphorylation of the receptors and the formation of docking sites for various STATs. The activated JAKs phosphorylate (P) and activate STATs. Some JAKs are associated with many different cytokine receptors, but JAK3 associates with only IL2, predominantly to its gamma chain subunit. The STAT family consists of six members: STAT1, STAT2, STAT3, STAT4, STAT5 and STAT6, which upon phosphorylation dissociate from the receptor to form homodimers or heterodimers and translocate to the nucleus and bind to transcription factor binding sites on the target DNA to regulate transcription of immune and inflammatory genes including glial markers, e.g., GFAP, AIF1. AIF1, allograft inflammatory factor 1 (also known as IBA1, ionized calcium-binding adapter molecule 1); CNTF, ciliary neurotrophic factor; CSF2, colony stimulating factor 2; CSF3, colony stimulating factor 3; GFAP, glial fibrillary acidic protein; gp130, glycoprotein 130; IFNA, interferon alpha; IFNB, interferon beta; IFNG, interferon gamma; IFNR, interferon receptor; IL11, interleukin 11; IL15, interleukin 15; IL2, interleukin 2; IL21, interleukin 21; IL2R, interleukin 2 receptor; IL3, interleukin 3; IL3R/βC, interleukin 3 receptor beta chain subunit; IL4, interleukin 4; IL5, interleukin 5; IL6, interleukin 6; IL6R, interleukin 6 receptor; IL7, interleukin 7; IL9, interleukin 9; JAK1, janus kinase 1; JAK2, janus kinase 2; JAK3, janus kinase 3; JAK-STAT, janus kinase-signal transducer and activator of transcription; LIF, leukemia inhibitory factor; OSM, oncostatin M; P, phosphate group; STAT1, signal transducer and activator of transcription 1; STAT2, signal transducer and activator of transcription 2; STAT3, signal transducer and activator of transcription 3; STAT4, signal transducer and activator of transcription 4; STAT5, signal transducer and activator of transcription 5; STAT6, signal transducer and activator of transcription 6; TYK2, tyrosine kinase 2.

The JAK/STAT signaling cascade is one of the critical communication pathways pivots in orchestrating several cellular processes, including the immune function. The JAK/STAT pathway (Figure 7) constitutes a rapid transfer of signals from cell-membrane receptors to the nucleus and induces the expression of various essential mediators of inflammation [377,378], as well as of innate and adaptive immunity [379]. Thus, the JAK/STAT pathway serves as a vital downstream mediator for a myriad of cytokines, hormones, and growth factors [4,37,316]; hematopoiesis, adipogenesis, inflammation, and apoptosis [378]. Extracellular factors, e.g., leptin and growth hormone also affect JAK/STAT signaling and control gene expression [4].

Ligands activate receptors linked to JAK/STAT and initiate the process of phosphorylation. Phosphorylated JAKs proceed towards activating several substrates and provide docking sites for STATs, which in turn become phosphorylated and exhibit the full spectrum of STAT activity [380]. During this signaling process, phosphorylated STATs are released from the receptor complex, undergo dimerization through -SH2 domain interactions, and translocate into the nucleus. Therefore, activating the receptors associated with JAKs is critical to initiate the JAK trans-phosphorylation and subsequent recruitment of one or more STATs to be phosphorylated [380].

In general, STATs often regulate transcription through the following sequential mechanisms: (a) first, STAT is interlinked with the N-terminal coiled-coil DNA binding site linker Src-homology 2 (SH2) and C-terminal to facilitate transcription activation, (b) second, transcriptional complexes are formed between STAT and other non-STAT transcription factors to initiate transcription, (c) third, STAT binds to non-STAT DNA binding domain to augment STAT-mediated transcription, and (d) fourth, STAT and non-STAT transcription factors bind to discrete DNA binding sites and synergistically coordinate transcription [378].

The cytoplasmic JAK family comprises four isoforms, JAK1, JAK2, JAK3, and TYK2 [37,380], whereas STAT has been classified into seven isoforms including STAT1, STAT2, STAT3, STAT4, STAT5A, STAT5B and STAT6 [381]. Overall, dysregulation of JAK/STAT activity is linked with inflammatory, autoimmune, cancer, and metabolic diseases [4].

It has been well accepted that activation of STAT3 is regulated by post-translational modification, particularly by phosphorylation at the serine and tyrosine residues. Selectively blocking the JAK/STAT and NF-κB pathways have been shown to reduce microgliosis cell death [382]. The JAK/STAT pathway has been shown to be linked to astrocyte differentiation, glial activation, and glial gene responses upon neuronal injury [37,316]. These early studies showed the target genes of STAT3 constitute cytokines and growth factors (IL6; interleukin 10/IL10; interleukin 6 cytokine family signal transducer/IL6ST or gp130; oncostatin M/OSM), apoptosis-related genes (BCL2 apoptosis regulator/BCL2; BCL2-Like 1/BCL2L1 or BCL-XL), acute phase response factors (C-reactive protein/CRP; alpha2-macroglobulin/A2M), transcription factors (Fos proto-oncogene, AP-1 transcription factor subunit/FOS or C-FOS; Jun proto-oncogene, AP-1 transcription factor subunit/JUN or C-JUN), and cell cycle-related genes (cyclin-dependent kinase inhibitor 1A/CDKN1A or p21; cyclin D1/CCND1; cyclin E1/CCNE1).

A crucial function for STAT3 in regulating GFAP expression has been identified during astrogenesis and astrogliosis, in which signaling molecules like cytokines act via the IL6/gp130-receptor to activate the JAK/STAT and MAPK pathways [316,383]. Following an injury to the striatal dopaminergic nerve terminals mediated by the dopaminergic neurotoxicant, 1-methyl-4-phenyl-1,2,3,6-tetrahydropyridine (MPTP), the JAK2/STAT3 pathway is specifically activated to upregulate GFAP expression in striatal astrocytes [316].

Altered JAK2/STAT3 signaling is involved in several neurodegenerative diseases and appears to be independent of an inflammatory event [384]. Inhibiting the JAK/STAT3 pathway has been shown to increase the frequency of Huntingtin protein aggregates in reactive astrocytes, a pathological hallmark of HD [385]. In addition, STAT3, STAT4, and TYK2 genetic variants have been associated with MS susceptibility [386]. Taken together, the JAK/STAT pathway is critical for cytokine-mediated signal transduction associated with a multitude of functions such as cellular proliferation, differentiation, and immune homeostasis.

### 4.4. Activation of MAPK Pathway in Neural Injury and Gliosis

**Figure 8:**
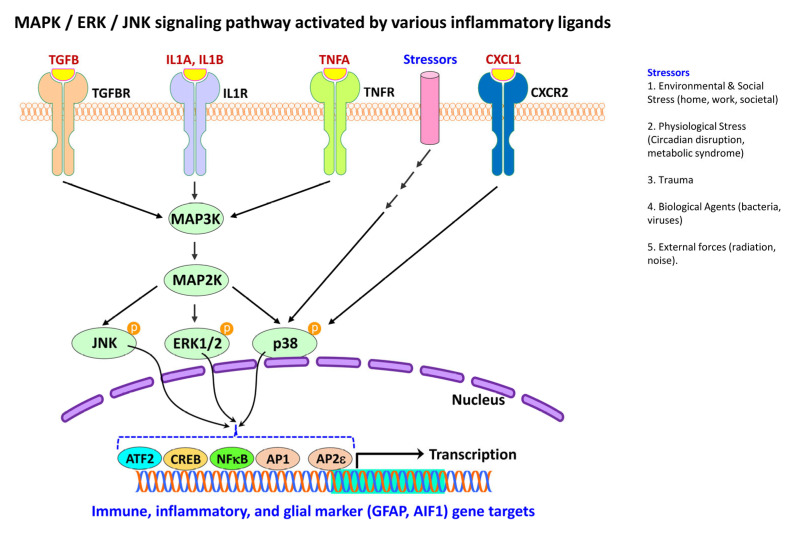
MAPK/ERK/JNK signaling in neuroinflammatory response. Schematic representation depicts MAP kinase activation by growth factors, stressor and cytokines which triggers a phosphorylation cascade. Agonist binding to TGFBR, IL1R, TNFR, stress receptors or CXCR2 leads to activation of the MAP3K, which has potential to activate various other MAP kinases by phosphorylating and activating the respective MAP2K. MAP2K has a restricted specificity for MAP kinase substrates. Upon activation, JNK, ERK1/2, and p38 further regulate gene transcription through activation and recruitment of transcription factors such as ATF2, CREB, NFkB, AP1 and AP2ε to the nucleus to regulate transcription of immune and inflammatory genes including glial markers, e.g., GFAP and AIF1. Arrows indicate the activation process in the signaling cascade and multiple arrows indicate several intermediates in the signaling cascade. AIF1, allograft inflammatory factor 1 (also known as IBA1, ionized calcium-binding adapter molecule 1); AP1, activator protein 1 (also known as JUN, Jun protooncogene AP1 transcription factor subunit or V-Jun avian sarcoma virus 17 oncogene homolog); AP2ε, transcription factor AP 2 epsilon (also known as TFAP2E); ATF2, activating transcription factor 2; CREB, cyclic AMP response element binding protein; CXCL1, C-X-C motif chemokine ligand 1; CXCR2, C-X-C motif chemokine receptor 2; ERK 1/2, extracellular signal-regulated kinase 1 and 2; GFAP, glial fibrillary acidic protein; IL1A, interleukin 1alpha; IL1B, interleukin 1 beta; IL1R, interleukin 1 receptor; JNK, c-Jun N-terminal kinase; MAP2K, mitogen activated protein kinase kinase; MAP3K, mitogen activated protein kinase kinase kinase; NFkB, nuclear factor kappa-B; P, phosphate group; p38, p38 mitogen activated protein kinase (p38 MAPK); TGFB, transforming growth factor beta; TGFBR, transforming growth factor beta receptor; TNFA, tumor necrosis factor alpha; TNFR, tumor necrosis factor receptor.

The MAPK pathway is a major signaling cascade, which regulates a diverse feature of cellular processes such as initiation, propagation, proliferation, differentiation, and apoptosis. The MAPK family (Figure 8) are serine/threonine kinases that direct cellular responses arising from various stimuli including growth factors, extracellular matrix proteins, inflammatory mediators and stressors [4,387].

In eukaryotic cells, at least three distinct types of MAPK cascades are known. These include mitogen-activated protein kinases 1 and 2 (MAPK3/ERK1 and MAPK1/ERK2; also referred to as p42/44 MAPK or p42/44 ERK), mitogen-activated protein kinase 8 (MAPK8; also referred to as c-Jun N-terminal kinase/JNK or stress-activated protein kinase/SAPK), and mitogen-activated protein kinase 14 (MAPK14; also referred to as p38 MAPK) signal transduction pathways [10]. ERK has been shown to be activated by mitogens and differentiation signals [4], and the ERK/MAPK signaling pathway forms the core signaling network for regulating cellular development, growth, and division. The ribosomal protein S6 kinase A1 (RSK) is a downstream effector of the ERK/MAPK signaling cascade [388].

JNK signaling cascade is widely involved in multiple physiological processes and mediates its actions through three alternate splicing isoforms, JNK α, β, and γ [389]. JNK activation is also associated with oncogene transformation growth factor-mediated signaling. Because inflammatory stimuli and stress activate JNK [4], it has been implicated in apoptosis, survival signaling, cell differentiation, and cytochrome *c* release from mitochondria.

Mitogen-activated protein kinase 14 (MAPK14; also known as p38 MAPK) is activated by toxicants, UV irradiation, heat shock, osmotic stress, proinflammatory cytokines, lipopolysaccharides, protein synthesis inhibitors, and certain mitogens [389]. It has been shown that the specific isoform p38δ is critical for interferon signaling, where it directs the phosphorylation and activation of phospholipase A2 in the cytoplasm. IFNA or IFNG-mediated activation of p38 MAPK also produces phosphorylation of the transcription factor STAT1 on Ser^727^ [389]. Collectively, activation of MAPKs, including ERK1/2, JNK, and p38 MAPK, leads to phosphorylation and activation of p38 transcriptions factors known to be abundant in cytoplasm or nucleus, that trigger the inflammatory responses in response to a variety of insults such as physical, chemical, biological, psychological, infectious, or microbial factors [4].

In post-mortem brain samples obtained from patients with AD, phosphorylated p38 is seen both in neurons and glia in the vicinity of the amyloid plaques [390]. However, in mouse models of AD, p38 MAPK is seen only within microglial cells [391]. Reactive astrocytes in the brains of patients with ALS have been shown to contain the active form of ERK, JNK, and p38 MAPK [385,392]. In mouse models of ALS, accumulation and activation of p38 MAPK is an essential feature to form hypertrophic astrocytes and reactive microglial state that accounts for the initiation and progression of motor neuron pathology [393].

## 5. GFAP Gene Expression as an Index of Astroglial Activation

The glial reaction to nervous system damage often termed gliosis, represents a hallmark of all types of neural injury [37,38]. Microglia and astrocytes are differentially involved in engulfing synapses at the sites of neuronal injury and reactive gliosis [394]. While the molecular events related to microglial activation following neuronal injury are limited, experimental studies have shown that like astroglia, activated microglia also serve as microsensors of brain injury, including that engendered by disease or toxicants [37,38]. Reactive astrogliosis is also seen in neurological disease states including neurodegeneration [385,395], epilepsy [396], ischemic stroke [397], demyelination [398,399], and traumatic brain injury [400,401].

When astrocytes react to injury, they undergo hypertrophy and upregulate the expression of GFAP. The basal levels of GFAP in astrocytes of different brain regions is variable [402,403]. For example, the hippocampal astrocytes display higher GFAP expression than the cortical or striatal astrocyte populations [404,405,406].

GFAP is a type-III intermediate filament and is the signature molecule among several intermediate filaments in astrocytes [407]. It is a relatively non-soluble acidic cytoskeletal protein. GFAP intermediate filament has a diameter of 10 nm, with the alpha-helical rod domain flanked by a flexible N-terminal tail domain [408]. The human GFAP consists of 432 amino acids with head and tail domains flanking a central α-helical rod domain encoded by a gene on chromosome 17q21 [409,410]. The gene consists of eight introns and nine exons, with four alternative exons and two alternative introns (3 kb, mRNA). A recent study demonstrated that mutations in the intron regions of the GFAP gene result in gene splicing and expression of various GFAP isoforms, aggregation, and accumulation of which leads to astrocyte dysfunction [411].

To date, six isoforms of GFAP have been identified [410,412] and classified as GFAP alpha (α), beta (β), gamma (γ), delta (δ), epsilon (ε), and kappa (κ). GFAPα is the major component of the astrocytic cytoskeleton and scaffolding required for the maintenance of its shape, structure, and plasticity. In humans, the promoter region of the GFAP gene extends from -2162 to +47, containing binding sites for several transcription factors [413]. The inhibition of the histone deacetylase activity causes a GFAPα to GFAPδ isoform shift mediated by serine and arginine-rich splicing factor 6 (SRSF6; also known as SR splicing factor 6/SR6) [414] resulting in downregulation of GFAP expression, which is suggestive of a regulatory function. The GFAP protein undergoes post-translational modifications via citrullination, glycosylation, lipoxidation, and phosphorylation, which influence its properties and function, including cytoskeletal assembly [412]. Experimental studies using rodent models and human cell lines have revealed that microRNAs (miRNA) regulate GFAP. However, direct regulation of the human GFAP gene by miRNA remains to be elucidated [413].

## 6. Concluding Remarks

Occupational brain injury and toxicant-induced neuronal damage are increasingly recognized to dysregulate the homeostatic signaling cascade in the healthy brain, causing gliosis and neuroinflammation. With the advent of new knowledge in the morphometric and cytokine field, the critical role of toxicant-induced acute or chronic neuroinflammation through gliosis is emerging, providing a foundation for understanding the pathophysiology of various neurological disorders. The polarization of microglia, release of inflammatory cytokines, microglial activation, reactive astrocytes, and high expression of GFAP provides robust evidence to the molecular underpinnings of neural injury and a benchmark for understanding various injury- or disease- models. Consistent with these findings, this review identifies and synthesizes the evidence about how a wide variety of ligands insult the homeostatic gene signature that could eventually trigger the pathological signaling cascades, including protein phosphorylation, NF-κB, JAK/STAT, and MAPK pathway, which can potentially sustain even after the cessation of the toxicant exposure or occupational injury.

Nevertheless, transient changes in the phenotype expression can be anticipated in the early phase of head injury, concussion, and TBI; however, the long-term phenotypic changes require a significant number of resident cells to change and secrete signaling molecules that sequentially activate various intermediate steps culminating in signal transduction and gene expression. In neurodegenerative diseases, understanding the specificity of the toxicant’s effect and their cytokine signature, along with any identifiable intermediate steps in the signaling cascades, can provide avenues for strategic therapeutic intervention.

Neurotoxicological studies aim to link the mechanistic processes within the cells and tissues to elucidate the physiological substrates of impaired functions associated with a toxicant. These substrates form a broad spectrum that reflects the biological organization hierarchy, bringing neuroinflammation into the context. At one end of the spectrum are the brain cells, including glia, and at the other is an organism exhibiting neurological disorders originating from the aftermath of gliosis. Cellular interactions in the brain compartment are becoming essential components of tissue functions. Microglia and astroglia exhibit an intimate association at the physical, molecular, and biochemical levels. They exist in proximity with neurons and endothelial cells of the BBB to maintain cellular interaction and transport of small molecules, which are critical for neuron-glia omnidirectional signaling primarily at the tripartite synapses.

This review has provided a comprehensive vision of recent advancements in the field to identify the possible targets as the result of occupational injury or the toxicant’s effect, bringing the neuroinflammation perspective into place for the implication of gliosis and subsequent progression of the neurodegenerative events in diseases including AD, PD, CJD, ALS, and MS. New research directions should include experimental models in which heterogeneous cell interactions are preserved during toxicant exposure in neuroinflammation arising from an occupational injury or toxicant-induced neuronal damage. In addition to in vivo assessment, a system of co-cultures, brain slices, and primary cultures prepared from the brains of toxicant-exposed animals should make it possible to design the strategy from the cell biology endpoint of the spectrum towards the tissue level, and from there, perhaps our view of the other end of the spectrum will be insightful.

## Figures and Tables

**Table 1:** Astroglial activation associated with occupational toxicants, brain injuries, and neurological disorders.

Toxicant/Injury/Disease	Brain Area(s) Affected	Reference(s)
*Occupational Toxicants:*		
1,1,1-trichloroethane	CTX, CER, HIP	[41]
Acrylamide	CER	[42]
Chlorpyrifos	STR	[43]
Cyclohexane	HIP	[44]
Dichloromethane	FCT, MCT	[45]
Fracking Sand Dust	OB, STR, CER	[46]
Manganese	GP, SN-R	[47]
Manganese	HIP	[48]
Oil dispersant (COREXIT EC9500A)	FCT, HIP	[49]
Silver nanoparticles	FCT, HIP	[50]
Toluene	HIP	[51]
Toluene	HIP, CER	[52]
Toluene	STR	[53]
Trimethyltin	HIP	[54]
Trimethyltin	HIP	[55]
Welding fumes	STR, MB	[56]
Welding fumes	STR, MB	[57]
Xylene	FCT, CER	[45]
*Brain Injuries:*		
Cerebellar stab injury	CER	[58]
Cortical stab injury	CTX	[59]
Cortical stab injury	CTX	[60]
Facial nerve lesion	CTX	[61]
Forebrain stab lesion	HIP	[62]
Hippocampal stab wound	HIP	[63]
Severe focal brain ischemia	CTX	[64]
Transient global ischemia	HIP	[65]
Transient global ischemia	HIP	[66]
Transient MCAO	STR, HIP, CTX	[67]
Traumatic brain injury	MCT	[68]
Traumatic brain injury	CTX	[69]
Traumatic brain injury	HIP	[70]
*Neurologic diseases/disorders:*		
Alzheimer’s disease	TCT	[71]
Alzheimer’s disease	HIP	[72]
Alzheimer’s disease	CN, THL, CER, BS	[73]
Alzheimer’s disease	HIP	[74]
Alzheimer’s disease	CTX, PUT, AMG	[75]
Alzheimer’s disease	HIP, FCT, PCT, TCT, CER	[76]
Alzheimer’s disease	ECT	[77]
Alzheimer’s-type dementia	TCT	[78]
Amyotrophic lateral sclerosis	CTX	[79]
Creutzfeldt-Jackob disease	CTX	[80]
Creutzfeldt-Jackob disease	OB, CTX, HIP, AMG	[81]
Multiple sclerosis	CTX	[82]
Multiple sclerosis	WM	[83]
Parkinson’s disease	SN, LC, DVN	[84]
Parkinson’s disease	OB	[85]
Schizophrenia with dementia	FCT, TCT	[86]

AMG, amygdala; BS, brainstem; CER, cerebellum; CN, cingulate nucleus; CTX, cortex; DVN, dorsal vagal nucleus; ECT, entorhinal cortex; FCT, frontal cortex; GP, globus pallidus; GP, globus pallidus; HIP, hippocampus; LC, locus coeruleus; MB, midbrain; MCT, motor cortex; OB, olfactory bulb; PCT, parietal cortex; PUT, putamen; SN, substantia nigra; SN-C, substantia nigra pars compacta; SN-R, substantia nigra pars reticulata; STR, striatum; TCT, temporal cortex; THL, thalamus; WM, white matter.

**Table 2:** Microglial activation associated with occupational toxicants, brain injuries, and neurological disorders.

Toxicant/Injury/Disease	Brain Area(s) Affected	Reference(s)
*Occupational Toxicants:*		
2, 5-Hexanedione	SN	[87]
Acrylamide	SN	[88]
Aluminum	STR, HIP, CTX	[89]
Cypermethrin	STR, SN	[90]
Diacetyl	OB	[91]
Dichlorvos	STR, SN	[92]
Diesel Exhaust	OB, CTX, MB, SN	[93]
Diethyldithiocarbamate	ECT, HIP, HYP	[94]
Iron oxide nanoparticles	OB, HIP, STR	[95]
Manganese	SN	[96]
Manganese	GP	[97]
Paraquat + Maneb	LC	[98]
Rotenone	STR, SN	[99]
Rotenone	HIP, CTX	[100]
Silver nanoparticles	OB	[101]
Silver nanoparticles	OB	[102]
Trimethyltin	HIP	[103]
Trimethyltin	HIP	[55]
Welding fumes	STR, MB	[104]
Welding fumes	STR, MB	[105]
*Brain Injuries:*		
Cortical stab injury	CTX	[60]
Mild focal brain ischemia	STR	[106]
Transient focal ischemia	SN	[107]
Transient global ischemia	STR, HIP	[108]
Transient MCAO	SN	[109]
Traumatic brain injury	CTX	[110]
Traumatic brain injury	CWM, THL	[111]
Traumatic brain injury	CTX	[69]
Traumatic brain injury	HIP	[70]
*Neurologic diseases/disorders:*		
Alzheimer’s disease	FCT, TCT, OCT, CER, BS	[112]
Alzheimer’s disease	FCT	[113]
Alzheimer’s disease	ITG, MTG	[114]
Alzheimer’s disease	CER	[115]
Alzheimer’s disease	HIP	[116]
Alzheimer’s disease	FCT	[117]
Alzheimer-type dementia	ECT, CCT, TCT	[118]
Amyotrophic lateral sclerosis	CTX, HIP	[119]
Amyotrophic lateral sclerosis	FTCT	[120]
Amyotrophic lateral sclerosis	CC	[121]
Creutzfeldt-Jackob disease	FCT, TCT, STR, TH, CER	[122]
Multiple sclerosis	WM	[83]
Multiple system atrophy	STR	[123]
Parkinson’s disease	SN	[124]
Parkinson’s disease	SN	[125]
Viral Encephalitis	FCT, TCT, OCT, CER, BS	[112]
Wilson’s disease	FCT, TCT, OCT, CER, BS	[112]

BS, brainstem; CC, corpus callosum; CCT, cingulate cortex; CER, cerebellum; CTX, cortex; CWM, cerebral white matter; ECT, entorhinal cortex; FCT, frontal cortex; FTCT, frontotemporal cortex; GP, global pallidus; HIP, hippocampus; HYP, hypothalamus; ITG, inferior temporal gyrus; LC, locus coeruleus; MB, midbrain; MTG, middle temporal gyrus; OB, olfactory bulb; OCT; occipital cortex; SN, substantia nigra; STR, striatum; TCT, temporal cortex; THL, thalamus; WM, white matter.

**Table 3:** Glial inflammatory mediators associated with Alzheimer’s disease.

Glial Cell Type	Inflammatory Mediator	Cellular (Neural) Effect	Reference(s)
M1 Microglia (reactive) Astrocyte (reactive)	TNFA	↑ Glutaminase ↑ Glutamic acid ↑ NO ↑ Peroxynitrite formation ↑ BACE 1 & 2 (Beta Secretases) ↑ APH1A (Gamma secretase) ↑ Aβ formation ↑ Aβ aggregation ↑ Aβ oligomers ↑ Aβ fibrils/plaques ↑ Aβ toxicity ↑ Tau ↑ P-Tau ↑ P-Tau ↑ P-Tau aggregates ↑ Tau fibrils/NFTs ↑ Synaptic excitotoxicity ↑ Synaptic dysfunction ↑ Neuronal Apoptosis ↓ Aβ clearance ↓ LTP	[245,246,247,248,249,250,251]
M1 Microglia (reactive) Astrocyte (reactive)	IL1B	↑ APP expression ↑ Aβ formation ↑ Aβ aggregation ↑ Aβ oligomers ↑ Aβ fibrils/plaques ↑ Aβ toxicity ↑ Tau ↑ P-Tau ↑ P-Tau ↑ P-Tau aggregates ↑ Tau fibrils/NFTs ↑ NO ↑ Peroxynitrite formation ↑ S100B ↑ p38 MAPK ↑ PTGS2 (COX2) ↑ Prostaglandin E2 (PGE2) ↑ Synaptic plasticity ↑ Synaptic dysfunction ↑ Neuronal damage ↑ Neuronal death ↓ LTP	[245,247,252,253,254,255,256,257,258,259,260]
M1 Microglia (reactive) Astrocyte (reactive)	IL6	↑ APP expression ↑ Tau ↑ P-Tau ↑ P-Tau ↑ P-Tau aggregates ↑ Tau fibrils/NFTs ↑ p38 MAPK ↑ p35 activator ↑ CDK5 ↓ LTP	[261,262,263,264,265]
M1 Microglia (reactive) Astrocyte (reactive)	IL18	↑ Aβ formation ↑ Aβ aggregation ↑ Aβ oligomers ↑ Aβ fibrils/plaques ↑ Aβ toxicity ↑ Tau ↑ P-Tau ↑ P-Tau ↑ P-Tau aggregates ↑ Tau fibrils/NFTs ↑ Synaptic dysfunction ↑ Neuronal damage ↑ Neuronal death ↓ LTP	[266,267,268]
M1 Microglia (reactive) Astrocyte (reactive)	COX1	↑ Inflammation	[269]
M1 Microglia (reactive) Astrocyte (reactive)	NO	↑ Inflammation ↑ Protein oxidative damage ↑ Lipid oxidation ↑ Tau fibrils/NFTs ↑ Synaptic excitotoxicity ↑ Synaptic dysfunction ↑ Neuronal damage ↑ Neuronal death	[270,271,272]

**Table 4:** Glial inflammatory mediators associated with Parkinson’s disease.

Glial Cell Type	Inflammatory Mediator	Cellular (Neural) Effect	Reference(s)
M1 Microglia (reactive) Astrocyte (reactive)	TNFA	↑ SNCA fibrils ↑ SNCA aggregation ↑ Excitatory synaptic transmission ↑ AMPA receptors ↑ Synaptic dysfunction ↑ Neuronal apoptosis ↑ Neuronal degeneration ↑ mEPSC frequency ↑ mEPSC amplitude ↓ LTP ↓ Inhibitory synaptic transmission ↓ GABRA1 receptors ↓ mIPSC amplitude	[291,292,293,294,295,296,297,298]
M1 Microglia (reactive) Astrocyte (reactive)	IL1B	↑ MAPK ↑ SRC Kinase ↑ SNCA fibrils ↑ Apoptosis ↑ Excitotoxicity ↑ Synaptic dysfunction ↑ Neuronal damage ↑ Neuronal death	[299,300,301,302,303]
M1 Microglia (reactive) Astrocyte (reactive)	TGFB	↑ Deregulation of ligands ↑ Deregulation of receptors ↑ Neuronal damage ↑ Neuronal death	[304,305,306,307]
M1 Microglia (reactive) Astrocyte (reactive)	NO	↑ Peroxynitrite formation ↑ S-Nitrosylation of proteins ↑ S-Nitrosylation of PARK proteins ↑ Excitotoxicity ↑ Impaired mitochondria ↑ Mitochondrial dysfunction ↑ Neuronal damage ↑ Neuronal death	[308,309,310,311,312]

mEPSCs, miniature excitatory postsynaptic currents; mIPSCs, miniature inhibitory postsynaptic currents.

## Data Availability

Data sharing is not applicable to this review article as no datasets were generated or analyzed.
